# Outdoor air pollutants and asthma risk in adolescents: evidence from a systematic review and meta-analysis

**DOI:** 10.3389/fpubh.2025.1721233

**Published:** 2025-12-10

**Authors:** Wenwen Shi, Supapon Kaewsanmung, Wuttipat Kiratipaisarl, Ratana Sapbamrer

**Affiliations:** 1 Department of Community Medicine, Faculty of Medicine, Chiang Mai University, Chiang Mai, Thailand; 2 Department of Nursing, Youjiang Medical University for Nationalities, Baise City, Guangxi, China; 3 Department of Physical Therapy, School of Integrative Medicine, Mae Fah Luang University, Chiang Rai, Thailand; 4 Environmental and Occupational Medicine Excellence Center (EnOMEC), Faculty of Medicine, Chiang Mai University, Chiang Mai, Thailand

**Keywords:** outdoor air pollution, asthma, adolescents, respiratory disease, particulate matter

## Abstract

**Background:**

Existing epidemiological evidence linking outdoor air pollution to asthma incidence in adolescents remains inconclusive, due to methodological heterogeneity in study design, exposure assessment, and asthma case definitions.

**Methods:**

This systematic review and meta-analysis synthesized observational studies examining associations between outdoor air pollutants and adolescent asthma. Comprehensive searches of PubMed, Embase, and Scopus were conducted using Medical Subject Headings (MeSH) and free-text terms. Eligible studies reported quantitative estimates, odds ratio (OR), hazard ratio (HR), relative risk (RR), prevalence ratio (PR), incidence rate ratio (IRR), or prevalence odds ratio (POR), with corresponding 95% confidence interval (CI). Pooled estimates were calculated using inverse-variance weighted fixed- and random-effects models. Heterogeneity was assessed using Cochran’s *Q* and *I*^2^ statistics.

**Results:**

Of 51 eligible studies, 40 were incorporated into the meta-analysis. Statistically significant associations were identified for each 10 μg/m^3^ increase in nitrogen dioxide (NO₂) (adjusted odds ratio [aOR] = 1.18; 95% CI: 1.08–1.29) and ozone (O₃) (aOR = 1.01; 95% CI: 1.00–1.03), as well as per 1 ppm increase in carbon monoxide (CO) (aOR = 1.31; 95% CI: 1.08–1.53). Traffic-related air pollution (TRAP) was also significantly associated with an elevated risk (aOR = 1.15; 95% CI: 1.10–1.21). In contrast, the pooled estimate for particulate matter with diameter ≤ 2.5 micrometers (PM_2.5_), particulate matter with diameter ≤ 10 micrometers (PM₁₀), and sulfur dioxide (SO_2_) did not reach statistical significance, These non-significant results, however, were accompanied by considerable between-study heterogeneity (*I*^2^ = 32.3% for PM₂.₅, 76.5% for PM₁₀, and 53.6% for SO₂), reflecting substantial inconsistency across the included studies and precluding a definitive conclusion regarding the absence of an association. Following adjustment for potential publication bias, the association for NO₂ remained statistically significant (aOR = 1.21; 95% CI: 1.10–1.33). Conversely, the point estimate for PM_2.5_ shifted toward the null and remained non-significant (aOR = 0.89; 95% CI: 0.70–1.12), while SO₂ continued to show no significant association.

**Conclusion:**

This meta-analysis highlights significant associations between adolescent asthma and specific combustion-related air pollutants. Future research should prioritize standardized exposure metrics, life-course cohort designs, and multipollutant modeling to inform targeted prevention and public health strategies.

**Systematic review registration:**

https://www.crd.york.ac.uk/prospero/, identifier PROSPERO database (CRD42024622246).

## 1 Introduction

With an estimated 260 million cases globally in 2021 and a projection to reach 275 million by 2050, asthma poses a substantial and growing public health challenge worldwide (1). This chronic, complex, and heterogeneous respiratory disorder remains the most common chronic condition among adolescents globally (2). A recent meta-analysis conducted in 2025, synthesizing data from 164 population-based studies, estimated the global prevalence of childhood asthma at 10.2% [95% confidence interval (95% CI): 9.5–11.0] (3). Notable geographic disparities in adolescent asthma prevalence were identified, ranging from as low as 0.3% in India to as high as 29.9% in Spain (4). Asthma imposes a considerable and multifaceted burden, as evidenced by frequent emergency department visits, hospitalizations, school absenteeism, activity limitations, and reliance on long-term pharmacological therapy (1–4). These adverse outcomes can interfere with children’s development, lower their academic performance, affect their mental health, and reduce their quality of life in the long term (1–4). To tackle this global health problem, it is important to identify modifiable risk factors, especially common environmental exposures, in order to develop effective, evidence-based prevention strategies (5, 6).

Air pollution, a complex mixture of particulate matter, gaseous pollutants, and biological or chemical agents, has been causally linked to both the development and worsening of asthma, particularly due to variations in exposure across time and location (7). These variations arise from differences in pollutant sources, as outdoor air pollution mainly originates from fossil fuel combustion (e.g., traffic, industrial activities, power generation), industrial emissions, and natural events such as wildfires. Among these, particulate matter with diameter ≤ 2.5 micrometers (PM_2.5_), nitrogen dioxide (NO₂), and ozone (O₃) are considered the most significant in terms of health impact (8). Long-term exposure to outdoor air pollution can lead to respiratory irritation and increase the risk of chronic respiratory diseases, including asthma, particularly among vulnerable populations such as children. According to the World Health Organization (WHO) (9), over 99% of the global population lives in areas where air pollution levels exceed WHO air quality guidelines, and an estimated 4.2 million deaths each year are attributed to outdoor air pollution. These statistics highlight outdoor air pollution as a critical global public health crisis.

Adolescence represents a critical and vulnerable period in the human life course. During this stage, the respiratory system continues to develop, with lung function maturing into early adulthood (10), and the immune system undergoes significant changes, including the maturation of T helper type 1 (Th1) and type 2 (Th2) cell balance (11). Compared to adults, adolescents have higher minute ventilation relative to body weight, and their typical behaviors, such as increased outdoor activity and proximity to ground-level air, may result in greater exposure to outdoor air pollutants (12). In addition to causing acute respiratory symptoms and impaired lung function, both early and prolonged exposure to air pollution can significantly affect asthma onset, progression, treatment outcomes, and long-term respiratory health (13).

Although numerous epidemiological studies have investigated the association between outdoor air pollution and asthma, several important knowledge gaps remain, particularly in relation to adolescents. One key limitation is the lack of systematic evaluation of both independent and combined effects of multiple air pollutants. In real-world settings, adolescents are simultaneously exposed to various pollutants, which may exhibit collinearity and interact in synergistic, additive, or antagonistic ways. However, most prior studies have examined single pollutants or isolated environments, limiting their ability to capture the complexity of actual exposure scenarios (14–16). Another major gap is the inconsistency in identifying critical exposure windows. Existing studies vary widely in the timing of exposure assessed (e.g., intrauterine, infancy, preschool), making it unclear which developmental period is most sensitive to air pollution. In addition, substantial heterogeneity in study findings for the same pollutant complicates efforts to draw firm conclusions regarding its effect on asthma outcomes. A further challenge lies in the variability of exposure units and increments used across studies, with measurements reported in microgram per cubic meter (μg/m^3^), parts per million (ppm), or parts per billion (ppb), hindering comparability. These limitations highlight the need for a rigorous, comprehensive, and adolescent-focused systematic review and meta-analysis, which holds significant scientific and public health relevance.

This study systematically synthesizes global observational evidence, including cohort, case–control, and cross-sectional studies, to evaluate the association between exposure to specific outdoor air pollutants and asthma outcomes among adolescents aged 10–19 years. A meta-analysis was conducted to quantify the effect sizes of outdoor air pollutant exposure on asthma risk, and a dose–response relationship model was developed. Subgroup analyses were performed to explore potential effect modification by study design, exposure window, exposure duration, and asthma subtype, aiming to identify sources of heterogeneity. Risk of bias was assessed using standardized evaluation tools to determine the overall certainty of the evidence. The findings from this review provide important scientific support for revising policy standards such as the WHO global air quality guidelines, advancing precision environmental risk management in clinical settings, and informing the design of intervention trials targeting mixed exposure effects. Ultimately, this research contributes to an evidence-based foundation for reducing the burden of preventable respiratory diseases in adolescents and promoting health equity.

## 2 Methods

This systematic review was conducted in accordance with the *Cochrane Handbook for Systematic Reviews of Interventions* (17). The primary objectives were: (1) to systematically synthesize epidemiological evidence on the association between outdoor air pollution exposure and asthma, and (2) to conduct a quantitative meta-analysis of exposure–response relationships. The methodology followed the *Preferred Reporting Items for Systematic Review and Meta-Analysis Protocols* (PRISMA-P) and the *Meta-analysis of Observational Studies in Epidemiology* (MOOSE) guidelines (18, 19). The study protocol was prospectively registered in the PROSPERO database (CRD42024622246; 15 December 2023; https://www.crd.york.ac.uk/prospero/) and received ethical approval from the Research Ethics Committee, Faculty of Medicine, Chiang Mai University, Thailand (Approval No. Exemption 0391/2025, approved on 29 May 2025).

### 2.1 Data sources and search strategy

We conducted a systematic literature search across three electronic databases (PubMed, Embase, and Scopus) for articles. The search started on December 5, 2024, and the last search was on October 8, 2025. Search strategies employed Boolean operators to combine key terms, including “determinants,” “predictors,” “risk factors,” “outdoor air pollution,” “outdoor air quality,” “asthma,” “adolescent,” and “child.” To optimize retrieval sensitivity and specificity, we utilized Medical Subject Headings (MeSH) complemented by free-text terms in titles and abstracts. This approach enabled identification of synonymous and related terminology for keyword refinement. Database-specific controlled vocabularies (e.g., Emtree in Embase) and subject headings were systematically integrated into the search syntax.

### 2.2 Eligible criteria

Studies that met the following inclusion criteria were included in the study: (1) the timeframe from the establishment of the database to October 2025 was selected to capture the most recent body of evidence following the widespread adoption of key diagnostic technologies/relevant policy changes; (2) researches conducted as original observational studies (including cross-sectional studies, case–control studies, and cohort studies); (3) only full-text articles published in English were considered due to limitations in translation resources. A systematic search of gray literature was not performed, as this review focused exclusively on peer-reviewed primary research available in established academic databases; (4) focusing on postnatal outdoor air pollution exposure; (5) asthma identified as the outcome disease of the articles (both parent- or self-reported and clinically diagnosed); (6) the age of the studies population ranged from 10 to 19 years; (7) the association between exposure to outdoor air pollution and asthma was assessed; (8) data were analyzed by multivariable regression analysis; and (9) the results were interpreted using odds ratio (OR), hazard ratio (HR), relative risk (RR), prevalence ratio (PR), incidence rate ratio (IRR), or prevalence odds ratio (POR), and with a 95% CI.

### 2.3 Exposure and outcome classification

The primary exposure of interest was outdoor air pollution. Main outdoor air pollutants were as follows: (1) PM_2.5_; (2) particulate matter with diameter ≤10 micrometers (PM_10_); (3) carbon monoxide (CO); (4) NO₂; (5) O₃; (6) sulfur dioxide (SO_2_); (7) traffic–related air pollution (TRAP): the mixture of vehicle exhausts, secondary pollutants formed in the atmosphere, evaporative emissions from vehicles, and non-combustion emissions (e.g., road dust, tire wear) (20).

Exposure timing was categorized as: (1) long-term exposure (months to years, usually ≥1 year); (2) short-term exposure (hours to days, usually ≤14 days) (21). Exposure assessment includes: (1) questionnaire or interview; (2) environmental measurement/modeling (e.g., fixed site monitor and land used regression, LUR). The outcomes of interest were categorized as current or ever asthma. Current asthma was defined as a physician–diagnosed condition accompanied by prescribed asthma medication use or wheezing episodes within the preceding 12 months. Ever asthma referred to individuals previously diagnosed with asthma by a physician but not treated in the past year. Asthma outcomes were assessed via parent- or self–reported physician diagnosis or documented diagnosis in medical records.

### 2.4 Data extraction

All relevant studies were retrieved from the three selected databases and imported into EndNote X9 (Thomson Reuters, USA) for article management. This software was used to remove duplicates and facilitate the screening process. Two independent reviewers (W.W.S. and S.K.) screened the titles, abstracts, and full texts based on predefined inclusion criteria. A third reviewer (R.S.) was consulted to resolve any disagreements. For each eligible study, data were extracted on the following: first author, year of publication, study region, age of the study population, study design, sample size, exposure type, exposure duration, exposure assessment method (e.g., questionnaire or environmental measurement/modeling), asthma outcome, outcome assessment method (e.g., questionnaire or medical records), key findings, and adjusted confounders. Two reviewers (W.W.S. and S.K.) independently extracted and cross-verified the data. Adjusted odds ratio (aOR) was extracted as the primary measure of association.

### 2.5 Quality assessment

Study quality was assessed using the *National Heart, Lung, and Blood Institute* (NHLBI) quality assessment tools for observational cohort, cross-sectional, and case–control studies (22). These tools evaluate key methodological aspects related to internal validity. The cohort and cross-sectional tools include 14 items rated as “yes,” “no,” or “other” (e.g., cannot determine, not applicable, not reported), with overall quality classified as good (11–14 points), fair (6–10 points), or poor (1–5 points). The case–control tool comprises 12 items with the same rating system, and studies are similarly categorized as good (9–12 points), fair (5–8 points), or poor (1–4 points). Two independent reviewers (W.W.S. and W.K.) performed the quality assessments, and any disagreements were resolved through discussion with a third reviewer (R.S.) to reach consensus.

### 2.6 Data analysis

This study included various measures of effect size, such as OR, HR, RR, PR, and POR. To ensure consistency in the meta-analysis, all effect estimates were converted to OR, the most commonly used measure in this context. For studies reporting POR (23), conversions to OR were performed using established methods if possible (24–26). However, in several studies (15, 27–31), the necessary data, such as the number of exposed and unexposed individuals and asthma incidence rates, were not reported, making it impossible to derive OR directly. In these cases, the reported HR was retained. Where possible, HR was converted to RR and then to OR using established formulas (32, 33).


$$\mathrm{RR}=\frac{1-e^{\mathrm{HR}\times \ln(1-r)}}{r}$$

$$\mathrm{RR}=\frac{\text{OR}}{\left(1-r)+(r\times \text{OR}\right)}$$

In the four studies mentioned, the reported incidence of asthma was relatively low: 3.03, 0.64 and 1.59%, 1.42, and 7.2 and 6.3%, respectively (27–30). Given that all incidence rates were below 10%, the assumption of a rare outcome is applicable. Under this assumption, HR is considered approximately equivalent to RR and, by extension, to OR. To confirm this approximation, we converted HR to RR and subsequently to OR using established equations. As demonstrated in the forest plots, the converted OR closely aligned with the original HR, supporting the validity of this approach. Therefore, we used the OR derived from HR for inclusion in the meta-analysis. However, for three studies reported cumulative incidence of asthma was more than 10% (15, 23, 31), we reported POR and HR was retained.

To ensure comparability across studies, all effect estimates for outdoor air pollutants were standardized to a common exposure increment of 10 μg/m^3^. This was done by scaling the regression coefficients (*β*) and their standard errors (SE), originally reported per 1 μg/m^3^, by a factor of 10, and then applying exponential transformation using established equations (34). For studies reporting pollutant concentrations in ppb, specifically for NO₂, O₃, and SO₂, unit harmonization was performed using gas-specific conversion factors based on standard conditions (25 °C and 1 atm). The following multipliers were used: 1.881 for NO₂, 1.962 for O₃, and 2.619 for SO₂ (35). These converted values were then rescaled to a 10 μg/m^3^ increment using the same transformation equations (34). When studies did not provide sufficient information to perform these conversions, the original authors were contacted. If essential conversion parameters remained unavailable, the studies were excluded from the meta-analysis.


Beta=LN(RRo)/increment

SE=(LN(RRo_high)–LN(RRo_low))/(2×1.96×increment)

RRc=EXP(Beta×10)

RRc_low=EXP(Beta×10–1.96×SE×10)

RRc_high=EXP(Beta×10+1.96×SE×10)

RRo is the effect estimate originally reported in the paper with its low (RRo_low) and high (RRo_high) end of the CI; RRc is the estimate we converted to.

Heterogeneity across the included studies was assessed using Cochran’s *Q* test and the *I*^2^ statistic. *I*^2^ values were categorized into three levels: low (<25%), moderate (25–50%), and substantial (>50%), indicating increasing levels of variability between studies. For outcomes with low heterogeneity, a fixed-effect model was used, based on the assumption that effect sizes were similar across studies and that any variation was due to chance. In contrast, for outcomes showing moderate to substantial heterogeneity (*I*^2^ ≥ 25%), a random-effects model with inverse-variance weighting was applied, recognizing that the true effect sizes may vary across studies due to underlying differences in study populations, designs, or exposure assessment methods.

Subgroup analyses were conducted based on the type of air pollutant to explore potential effect modification. Sensitivity analyses were performed to examine differences across exposure duration, study design, asthma outcome type, and the choice between fixed-effect and random-effects models. To assess potential publication bias, funnel plots were generated, and Egger’s regression test was applied. The plots displayed effect size on the horizontal axis and sample size on the vertical axis to detect asymmetry indicative of small-study effects or publication bias. In cases where bias was identified, the trim-and-fill method was used to adjust the pooled estimates. All statistical analyses were performed using STATA software, version 17.0 (StataCorp LLC, College Station, TX, USA), with two-tailed tests and a significance level set at *p* value < 0.05.

## 3 Results

### 3.1 Study selection

**Figure 1:**
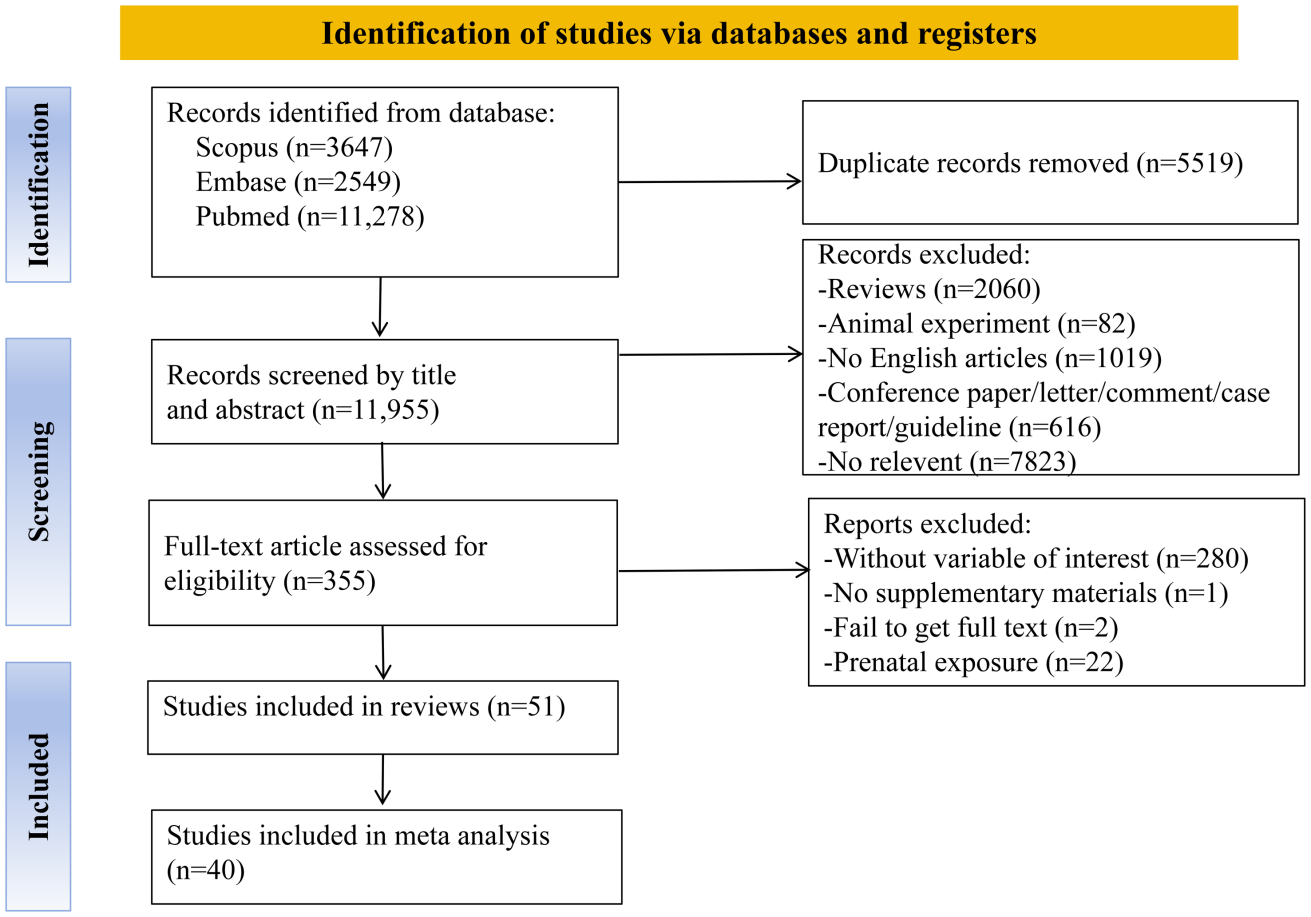
The PRISMA flow diagram of study selection.

The initial database search identified 17,474 records. After removing 5,519 duplicates, 11,955 unique records remained for title and abstract screening. From these, 355 articles were identified as potentially eligible and underwent full-text review based on predefined inclusion criteria. Ultimately, 51 studies met the criteria for inclusion in the systematic review, of which 40 were eligible for meta-analysis (Figure 1). Full details of the methodological transparency are provided in Supplementary Table S1.

### 3.2 The study characteristics regarding the association between outdoor air pollution and adolescent asthma

The 51 studies (15, 16, 23, 27–31, 36–78) included in this review, published between 1989 and 2025, comprised sample sizes ranging from 19 to 1,018,031 participants. Of these, 20 were cohort studies (prospective or retrospective) and 31 were cross-sectional studies. There was a notable geographic imbalance in study distribution. East Asia accounted for 21% of studies [China: 19% (*n* = 10); Japan: 2% (*n* = 1)], while North America contributed 20% [USA: 12% (*n* = 6); Canada: 6% (*n* = 3); Mexico: 2% (*n* = 1)]. Four studies (8%) were multi-country investigations, including two global-scale studies covering 30–50 countries. Limited evidence was available from Africa (4%, predominantly supranational analyses) and Eastern Europe, with 32% of countries represented by a single study. Exposure to outdoor air pollution was most commonly assessed through environmental monitoring or modeling approaches (*n* = 33). The primary health outcomes reported were current asthma and ever-diagnosed asthma. Detailed methodological characteristics and outcome data are presented in Table 1.

**Table 1:** The studies regarding the association between outdoor air pollutants and asthma.

Author, year (Region)	Age (years)	Study design	Sample size	Health outcome	Types	Findings POR/PR/OR/RR/HR/IRR (95%CI)	Confounders/covariates
Dockery et al., 1989 (USA) (36)	10–12	CO	5,422	Ever asthma ^a^	PM_2.5_^c, 1^	OR = 0.60 (0.30–1.40)	Gender, age, parental education, maternal smoking, gas stoves, and city of residence
					SO_2_^c, 1^	OR = 0.60 (0.30–1.20)	
					NO_2_^c, 1^	OR = 0.60 (0.30–0.90)	
					O_3_^c, 1^	OR = 1.90 (1.00–3.40)	
Duhme et al., 1998 (Germany) (23)	12–15	CS	6,852	Current asthma^a^	TRAP ^d^	POR = 1.06 (0.83–1.36) for seldom ^e^ (written questionnaire, *n* = 3,745)	Age, gender, study area, furry pets, active smoking in last month, environmental tobacco smoke exposure at home, mold or wet spots in bedroom, truck traffic, wood or coal heating, and parental atopy
						POR = 1.10 (0.90–1.35) for seldom ^e^ (video questionnaire, *n* = 3,745)	
						POR = 1.05 (0.63–1.73) for seldom ^e^ (written questionnaire, *n* = 3,107)	
						POR = 1.27 (0.82–1.97) for seldom ^e^ (video questionnaire, *n* = 3,107)	
						POR = 1.68 (1.28–2.21) for frequent or constant ^e^ (written questionnaire, *n* = 3,745) *	
						POR = 1.60 (1.26–2.02) for frequent or constant ^e^ (video questionnaire, *n* = 3,745) *	
						POR = 1.46 (0.88–2.41) for frequent or constant ^e^ (written questionnaire, *n* = 3,107)	
						POR = 1.72 (1.11–2.67) for frequent or constant ^e^ (video questionnaire, *n* = 3,107) *	
Wang et al., 1999 (China) (37)	11–16	CS	155,283	Current asthma^a^	PM_10_^c, 1^	OR = 1.00 (0.96–1.05)	Age, gender, resident area, and parents’ education level, exercise, smoking, alcohol, incense use, and environmental tobacco smoke exposure
					SO_2_^c, 1^	OR = 0.98 (0.95–1.02)	
					NO_2_^c, 1^	OR = 1.08 (1.04–1.13) *	
					CO ^c, 1^	OR = 1.15 (1.10–1.20) *	
					O_3_^c, 1^	OR = 1.11 (1.07–1.15) *	
Shima et al., 2000 (Japan) (38)	11–12	CO	840	Current asthma^a^	NO_2_^c, 1^	OR = 2.10 (1.10–4.75) *	Gender, history of allergic diseases, history of respiratory diseases under 2 years of age, feeding methods in infancy, parental history of allergic diseases, parent smoking habit, and use of unvented heat in the winter
Lin et al., 2001 (China) (39)	13–15	CS	1,018,031	Current asthma^a^	CO ^c, 1^	OR = 1.10 (1.03–1.16) for 0.75–0.99 ppm *	Gender, grade, family, passive smoking, incense burning, student exercise, and parental education level
						OR = 1.30 (1.18–1.42) for≧1.0 ppm *	
Kuo et al., 2002 (China) (40)	13–16	CS	12,926	Current asthma^a^	NO_2_^c, 1^	OR = 1.692 (1.155–2.480) for≧0.023 ppm *	Gender, age, residential area, parent’s education, number of smokers in family, number of cigarettes, use of incense smoke, and frequency of children’s activities
					SO_2_^c, 1^	OR = 1.198 (0.674–2.127) for≧0.005 ppm	
					O_3_^c, 1^	OR = 0.750 (0.318–1.769) for≧23 ppb	
					PM_10_^c, 1^	OR = 0.947 (0.640–1.401) for≧65.9 μg/m^3^	
Delfino et al., 2003 (USA) (41)	10–16	CO	19	Current asthma^a^	O_3_^c, 2^	OR = 0.60 (0.09–3.87) for 1-h max	Respiratory infections and temperature
						OR = 0.50 (0.08–3.23) for 8-h max	
					NO_2_^c, 2^	OR = 8.13 (1.52–43.4) for 1-h max *	
						OR = 7.14 (1.66–30.7) for 8-h max *	
					SO_2_^c, 2^	OR = 2.36 (1.16–4.81) for 1-h max *	
						OR = 1.91 (1.06–3.43) for 8-h max *	
					CO ^c, 2^	OR = 1.22 (0.43–3.43) for 1-h max	
						OR = 0.96 (0.27–3.38) for 8-h max	
Del-Rio-Navarro et al., 2006 (Mexico) (42)	13–14	CS	3,243	Current asthma^a^	TRAP ^d^	OR = 1.206 (1.066–1.363) for boys ^e^ *	Sneezing, runny, and blocked nose at any time and in the last 12 months, itchy rash at any time, weekly meat consumption, and smoking mother or babysitter
						OR = 1.136 (1.008–1.280) for girls ^e^ *	
Mi et al., 2006 (China) (43)	13–14	CS	1,414	Current asthma^a^	NO_2_^c, 2^	OR = 1.23 (0.87–1.73) for asthma attacks	Age, gender, smoking and observed water leakage and indoor molds in school
						OR = 1.44 (1.06–1.95) for asthma attacks and current asthma medication *	
					O_3_^c, 2^	OR = 0.37 (0.12–1.21) for asthma attacks	
						OR = 0.54 (0.21–1.41) for asthma attacks and current asthma medication	
Islam et al., 2007 (USA) (28)	17–18	CO	2,843	Ever asthma ^a^	PM_2.5_^c, 1^	HR = 0.65 (0.41–1.03) for low PM_2.5_ exposure over the 10th–90th percentile range FVC	Community, ethnicity, age, and gender
						HR = 0.46 (0.30–0.71) for low PM_2.5_ exposure over the 10th–90th percentile range FEV_1_ *	
						HR = 0.34 (0.21–0.56) for low PM_2.5_ exposure over the 10th–90th percentile range FEF_25–75_ *	
						HR = 1.41 (0.87–2.26) for high PM_2.5_ exposure over the 10th–90th percentile range FVC	
						HR = 1.08 (0.66–1.76) for high PM_2.5_ exposure over the 10th–90th percentile range FEV_1_	
						HR = 0.76 (0.45–1.26) for high PM_2.5_ exposure over the 10th–90th percentile range FEF_25–75_	
Solé et al., 2007 (Brazil) (44)	13–14	CS	16,209	Current asthma^a^	NO_2_^c, 1^	OR = 1.87 (1.47–2.38) for 81.0 μg/m^3^ (*n* = 3,181) *	Annual mean levels of O₃, CO, NO₂, and SO₂, socioeconomic status: infant mortality rate, poverty index, average nominal income for individuals older than 10 years, and specific environmental and population density characteristics of different cities
						OR = 0.93 (0.70–1.23) for 39.0 μg/m^3^ (*n* = 3,161)	
						OR = 0.97 (0.74–1.28) for 33.0 μg/m^3^ (*n* = 3,232)	
						OR = 1.57 (1.22–2.02) for 34.5 μg/m^3^ (*n* = 3,007) *	
					SO_2_^c, 1^	OR = 2.01 (1.56–2.60) for 7.0 μg/m^3^ (*n* = 3,181) *	
						OR = 1.04 (0.78–1.40) for 16.0 μg/m^3^ (*n* = 3,232)	
						OR = 1.08 (0.81–1.42) for 40.5 μg/m^3^ (*n* = 3,628)	
Ho et al., 2007 (China) (45)	10–17	CS	69,367	Current asthma^b^	CO ^c, 2^	OR = 1.984 (1.536–2.561) for female *	Age, rhinitis, eczema, born in city, county, parent’s education, exercise, smoking, drinking, rainy days, and temperature
						OR = 1.780 (1.377–2.302) for male *	
					O_3_^c, 2^	OR = 1.015 (1.001–1.029) for male *	
					PM_10_^c, 2^	OR = 0.993 (0.990–0.997) for female *	
Zhao et al., 2008 (China) (46)	11–15	CS	1,993	Current asthma^a^	SO_2_^c, 2^	OR = 0.97 (0.70–1.35)	Age, gender, parental asthma or allergy, passive smoking at home, home painting, and new floor and furniture in the last 12 month
					NO_2_^c, 2^	OR = 0.66 (0.37–1.10)	
					O_3_^c, 2^	OR = 0.50 (0.11–2.28)	
Brunekreef et al., 2009 (30 counries) (47)	13–14	CS	152,973	Ever asthma ^a^	TRAP ^d^	OR = 1.18 (1.08–1.28) for almost ^e^ *	Gender, region of the world, language, gross national income per capital, cooking fuel, maternal education, current maternal and paternal smoking, exercise, television viewing, consumption of fast food, current paracetamol use, and siblings
						OR = 1.08 (1.00–1.17) for frequent ^e^ *	
						OR = 1.01 (0.94–1.09) for seldom ^e^	
				Current asthma^a^	TRAP ^d^	OR = 1.53 (1.36–1.72) for almost ^e^ *	
						OR = 1.26 (1.13–1.41) for frequent ^e^ *	
						OR = 1.07 (0.97–1.18) for seldom ^e^	
Musharrafieh et al., 2009 (Canada) (48)	13–14	CS	3,115	Current asthma^a^	TRAP ^d^	OR = 0.90 (0.70–1.10) for continuously during daytime ^e^	Gender, nationality region, school, buses, trucks pass by house, passive smoking in household, asthma symptoms past year, rhinitis past year, and eczema past year
						OR = 1.30 (1.00–1.60) for all day long ^e^	
Sahsuvaroglu et al., 2009 (Canada) (49)	13–14	CS	29	Ever asthma ^a^	NO_2_^c, 1^	OR = 1.271 (0.992–1.627) for no hay fever girls *	Deprivation index and rate of repair
					PM_10_^c, 1^	OR = 1.044 (0.891–1.225) for no hay fever girls	
					SO_2_^c, 1^	OR = 1.246 (0.802–1.934) for no hay fever girls	
					O_3_^c, 1^	OR = 0.998 (0.691–1.440) for no hay fever girls	
Kasznia-Kocot et al., 2010 (Poland) (50)	13–15	CS	1,130	Ever asthma ^a^	TRAP ^d^	OR = 1.93 (1.09–3.41) for high density of road traffic *	Gender, wheezing last year, dyspnea with wheezing last year, doctor diagnosed asthma, maternal education, unemployment mother and father, the allergy and asthma of parents, parental smoking, birth body weight at least 3 months, breast feeding, nursery school attendance, kindergarten attendance, touch animals since birth, coal heating, carpets on floors, damp, stains, mold present at home 50-year-old building, and road traffic
Anderson et al., 2010 (50 countries) (51)	13–14	CS	322,529	Ever asthma ^a^	PM_10_^c, 1^	OR = 0.94 (0.87–1.01)	Gross national product per capital and allowing for clustering within country
Jerrett et al., 2011 (USA) (29)	10–18	CO	217	Ever asthma ^a^	NO_2_^c, 1^	HR = 1.29 (1.11–1.49) for in fall winter *	Hispanic ethnicity, enrollment group, medical insurance coverage, and community annual mean relative humidity
						HR = 1.27 (1.03–1.57) for in summer *	
						HR = 1.29 (1.07–1.56) for in annual *	
Cibella et al., 2011 (Italy) (52)	10–17	CS	2,150	Current asthma^a^	TRAP ^d^	OR = 1.84 (1.14–2.95) for frequent or constant of trucks passing on the street of residence on weekdays *	Gender, age, height, weight, BMI, subjects with≧one positive skin test, mold, dampness, eczema, parental asthma, environmental tobacco smoke, and rhinoconjunctivitis
Gonzalez-barcala et al., 2013 (Spain) (16)	13–14	CS	7,295	Ever asthma ^a^	TRAP ^d^	OR = 1.09 (0.82–1.44) for boys seldom ^e^	BMI, parental smoking and maternal education
						OR = 1.13 (0.83–1.52) for boys frequent ^e^	
						OR = 1.01 (0.64–1.60) for boys constant ^e^	
						OR = 1.30 (0.91–1.87) for girls seldom ^e^	
						OR = 1.09 (0.75–1.59) for girls frequent ^e^	
						OR = 1.18 (0.71–1.97) for girls constant ^e^	
				Current asthma^a^	TRAP ^d^	OR = 0.89 (0.64–1.25) for boys seldom ^e^	
						OR = 1.01 (0.71–1.45) for boys frequent ^e^	
						OR = 0.98 (0.57–1.69) for boys constant ^e^	
						OR = 1.02 (0.70–1.48) for girl seldom ^e^	
						OR = 1.12 (0.76–1.65) for girls frequent ^e^	
						OR = 1.17 (0.69–1.99) for girls constant ^e^	
Gruzieva et al., 2013 (Sweden) (53)	12	CO	3,633	Current asthma^a^	PM_10_^c, 1^	OR = 1.96 (1.08–3.53) for road traffic during the first year of life *	Municipality, social economic status, year the house was built, and heredity
						OR = 1.02 (0.68–1.54) for road traffic during since the previous follow-up	
Oluwole et al., 2013 (Nigeria) (54)	13–14	CS	1,736	Ever asthma ^a^	TRAP ^d^	OR = 1.01 (0.91–1.68) for seldom ^e^	Area of residence, home fuel, cat in home in past year, active smoking status, parent smoking, and siblings
						OR = 1.77 (1.04–3.01) for frequent ^e^	
						OR = 0.94 (0.54–1.63) for almost whole day ^e^	
Fuertes et al., 2013 (Europe) (55)	10	CO	5,078	Current asthma^a^	NO_2_^c, 1^	OR = 0.89 (0.73–1.08)	Gender, age, parental history of atopy, parental education, siblings, maternal smoking during pregnancy, smoke exposure in home, furry pets, use of gas stove for cooking, home dampness or indoor mold, intervention participation, cohort, and area
					PM_2.5_^c, 1^	OR = 0.97 (0.59–1.58)	
					O_3_^c, 1^	OR = 1.20 (0.98–1.48)	
Mölter et al., 2014 (UK) (56)	11	CO	927	Current asthma^a^	PM_10_^c, 1^ NO_2_^c, 1^	OR = 0.87 (0.55–1.38) OR = 1.05 (0.87–1.25)	Gender, age, body mass index, paternal income at birth, sensitization, family history of asthma, hospitalization during the first 2 years of life, and smoking within the child’s home during the first year of life
Gomes de Luna et al., 2015 (Brazil) (57)	13–14	CS	3,015	Current asthma^a^	TRAP ^d^	OR = 1.41 (0.79–2.52) for traffic of trucks/bus on the street often or almost every day	Rhinoconjunctivitis, rhinitis, fruits, fried snacks, stuffed biscuits, meat, vegetables, fast food, soft drinks, paracetamol ≧ once per month in last 12 months, physical activity, school type, and maternal education
Gehring et al., 2015 (Germany, Sweden, and Netherlands) (58)	14–16	CO	14,126	Ever asthma ^a^	NO_2_^c, 1^	OR = 1.13 (1.02–1.25) for birth address exposure *	Gender, maternal and paternal asthma and hay fever, native nationality, parental education, breastfeeding, older siblings, day-care attendance, maternal smoking during pregnancy, parental smoking at home, mold/dampness at home, pets, use of gas for cooking, and municipality
						OR = 1.03 (0.88–1.19) for current address exposure	
					PM_2.5_^c, 1^	OR = 1.25 (0.94–1.66) for birth address exposure	
						OR = 1.13 (0.85–1.49) for current address exposure	
					PM_10_^c, 1^	OR = 1.08 (0.77–1.51) for birth address exposure	
						OR = 0.91 (0.75–1.11) for current address exposure	
				Current asthma^a^	NO_2_^c, 1^	OR = 1.06 (0.88–1.26) for birth address exposure	
						OR = 1.04 (0.93–1.16) for current address exposure	
					PM_2.5_^c, 1^	OR = 1.34 (1.00–1.79) for birth address exposure *	
						OR = 1.18 (0.91–1.53) for current address exposure	
					PM_10_^c, 1^	OR = 1.10 (0.74–1.63) for birth address exposure	
						OR = 1.03 (0.80–1.34) for current address exposure	
Hedman et al., 2015 (Sweden) (30)	12–19	CO	2,747	Ever asthma ^a^	TRAP ^d^	HR = 1.10 (0.81–1.50) for living within 200 m from a heavily trafficked road or much used bus stop	Gender, parental history of asthma, ever smoking, number of siblings, ever cat, house dampness, maternal smoke, living place, allergic sensitization, weight, height, body mass index, and respiratory infections
				Current asthma^a^	TRAP ^d^	HR = 1.07 (0.75–1.53) for living within 200 m from a heavily trafficked road or much used bus stop	
Chiang et al., 2016 (China) (15)	11–14	CO	587	Ever asthma ^b^	TRAP ^d^	HR = 1.23 (0.87–1.73) for in 1999–2010 ^e^	Age, gender, smoking, alcohol consumption, living near roads, passive smoking, and indoor environmental factors (e.g., incense burning, mosquito incense burning, carpets, dehumidifiers, gas cookers, and gas tea kits)
					SO_2_^c, 1^	HR = 1.29 (0.91–1.83) for high level in 1999–2010	
Bowatte et al., 2016 (Australia) (59)	12, 18	CO	620	Current asthma^a^	TRAP ^c^	OR = 1.02 (0.86–1.22) for lengths of major roads in 150 m buffer of residence during 1 yr. of life in 12-year child	Parent asthma and smoking.
						OR = 1.02 (0.86–1.21) for lengths of major roads in 150 m buffer of residence during 1 yr. of life in 18-year child	
						OR = 0.78 (0.36–1.70) for living ≤150 m from a freeway or highway during 1 yr. of life in 12-year child	
						OR = 1.24 (0.65–2.36) for living ≤150 m from a freeway or highway during 1 yr. of life in 18-year child	
Rosa et al., 2016 (Italy) (60)	11–14	CS	410	Ever asthma ^a^	PM_10_^c, 2^	OR = 1.12 (1.00–1.21) *	Maternal asthma, child’s gender, child’s age, and socioeconomic status
Yang et al., 2016 (Netherlands) (61)	14	CO	3,701	Current asthma^a^	NO_2_^c, 1^	OR = 1.08 (0.97–1.21)	Gender, maternal education, parental allergies, breastfeeding, maternal smoking during pregnancy, pets, mold/ dampness in home, gas for cooking, daycare attendance during first year of life, and neighborhood percentage of low-income households
					PM_2.5_^c, 1^	OR = 1.02 (0.87–1.18)	
Greenberg et al., 2016 (Israel) (62)	17	CO	137,040	Ever asthma ^a^	NO_2_^c, 1^	OR = 1.301 (1.187–1.426) for 14.1–27.2 μg/m^3^	Birth country, birth year, body mass index, cognitive abilities, education, number of children, and social economic status
						OR = 1.391 (1.264–1.531) for 27.2–43.2 μg/m^3^	
					SO_2_^c, 1^	OR = 1.070 (1.016–1.126) for 6.7–13.3 μg/m^3^	
						OR = 1.369 (1.266–1.481) for 13.3–592.7 μg/m^3^	
Greenberg et al., 2017 (Israel) (63)	17	CS	137,040	Ever asthma ^a^	NO_2_^c, 1^	OR = 1.01 (1.01–1.01) for arithmetic mean of average concentration	Body mass index, country of birth, year of birth, cognitive abilities, education, number of siblings, and social economic status
					SO_2_^c, 1^	OR = 1.00 (1.00–1.00) for arithmetic mean	
Arrais et al., 2017 (Angola) (64)	13–14	CS	3,128	Current asthma^a^	TRAP ^d^	OR = 1.236 (0.85–1.79) for seldom ^e^	Rhinitis in the last 12 months, eczema ever, cooking fuel used at home, indoor home cooling system, frequency of paracetamol intake, number of siblings, pet, body mass index, and smoking at home
						OR = 1.36 (0.94–1.97) for frequently in the day ^e^	
						OR = 1.56 (1.05–2.34) for almost the whole day ^e^ *	
Skrzypek et al., 2019 (Poland) (65)	13–15	CS	936	Ever asthma ^a^	TRAP ^d^	OR = 2.16 (1.12–4.15) for living in the vicinity of a main road *	Gender, body mass index, maternal employment, exposure to environmental tobacco smoke at home, type of heating, traces of moisture or mold in the place of residence, and parental allergy
						OR = 2.31 (1.22–4.39) for traffic intensity near the place of residence *	
He et al., 2019 (China) (66)	17.5	CO	2,942	Ever asthma ^a^	NO_2_^c, 1^	OR = 1.03 (0.96–1.09) for in 0–2 year exposure	Gender, neighborhood income, household income, mother’s migration status, highest parental education level, family history of asthma, eczema, allergic rhinitis, and other air pollutants exposure
						OR = 1.01 (0.94–1.08) for in 3-8 year exposure	
					SO_2_^c, 1^	OR = 0.96 (0.89–1.03) for in 0–2 year exposure	
						OR = 0.98 (0.91–1.05) for in 3-8 year exposure	
					PM_10_^c, 1^	OR = 0.95 (0.89–1.02) for in 0–2 year exposure	
						OR = 0.95 (0.88–1.02) for in 3-8 year exposure	
Liu et al., 2020 (China) (67)	12–17	CS	22,574	Ever asthma ^a^	PM_2.5_^c, 1^	OR = 1.70 (1.45–2.01) *	Age, gender, obesity, birth weight, premature birth, breastfeeding, exercise time/week, area of residence per person, household income, parental education, passive smoking, family history of asthma, and average temperature during investigation and districts
					PM_10_^c, 1^	OR = 1.60 (1.38–1.86) *	
					NO_2_^c, 1^	OR = 1.58 (1.36–1.84) *	
				Current asthma^a^	PM_2.5_^c, 1^	OR = 1.72 (1.37–2.15) *	
					PM_10_^c, 1^	OR = 1.65 (1.34–2.03) *	
					NO_2_^c, 1^	OR = 1.64 (1.34–2.02) *	
To et al., 2020 (Canada) (68)	17	CO	1,286	Ever asthma ^b^	NO_2_^c, 1^	OR = 1.17 (1.05–1.31) for at birth exposure	Age, gender, parental education, income adequacy, number of people at home, birthweight, breastfeeding, enrollment in childcare, born within 3 weeks of due date, damp spots, gas to cook/heat, pets, roaches, mold, environmental tobacco smoke, and parental history of asthma and atopy
						OR = 1.12 (1.00–1.26) for at 3-year exposure	
					O_3_^c, 1^	OR = 1.22 (1.04–1.43) for at birth exposure	
						OR = 1.13 (0.97–1.31) for at 3-year exposure	
					PM_2.5_^c, 1^	OR = 0.82 (0.69–0.97) for at birth exposure	
						OR = 0.92 (0.81–1.04) for at 3-year exposure	
Rutter et al., 2020 (Multi-country) (69)	13–14	CS	224,436	Current asthma^a^	TRAP ^d^	OR = 1.14 (1.09–1.18) for heavy truck traffic *	Gender, mothers level of education, number of siblings, the current exposure of heavy truck traffic, fast food, television, paternal tobacco, maternal tobacco, and paracetamol
Kuiper et al., 2021 (Europe, Spain, Australia) (70)	10–18	CO	3,428	Current asthma^a^	NO_2_^c, 1^	OR = 1.29 (1.02–1.63) *	Age, gender, parental education, and parental asthma
					PM_2.5_^c, 1^	OR = 1.45 (0.90–2.36)	
					PM_10_^c, 1^	OR = 1.90 (1.06–3.41) *	
					O_3_^c, 1^	OR = 2.00 (1.11–3.58) *	
Radhakrishnan et al., 2021 (Ontario) (27)	10	CO	114,427	Ever asthma ^b^	NO_2_^c, 1^	HR = 0.70 (0.62–0.81) for Windsor region	Maternal age, maternal asthma, gender, rural geography, neighborhood material deprivation, cesarean delivery, neighborhood ethnic concentration, and fiscal year
						HR = 0.65 (0.60–0.69) for London Middlesex region	
					O_3_^c, 1^	HR = 0.72 (0.67–0.78) for Windsor region	
						HR = 0.65 (0.60–0.69) for London Middlesex region	
					SO_2_^c, 1^	HR = 0.80 (0.68–0.93) for Windsor region	
						HR = 0.81 (0.60–1.09) for London Middlesex region	
					PM_2.5_^c, 1^	HR = 0.75 (0.69–0.82) for Windsor region	
						HR = 0.71 (0.63–0.81) for London Middlesex region	
Ahmetaj et al., 2023 (Kosovo) (71)	13–14	CS	6,682	Current asthma^a^	TRAP ^d^	OR = 0.84 (0.16–4.51) for seldom in Ferizaj ^e^	Gender, exercise, television-watching, computer use, tablet use, smart phone use, siblings, paracetamol intake last year, current cat dog, and weight
						OR = 1.64 (0.30–8.90) for frequently in Ferizaj ^e^	
						OR = 1.35 (0.23–7.92) for almost in Ferizaj ^e^	
						OR = 2.41 (0.52–11.2) for seldom in Gjakova ^e^	
						OR = 3.27 (0.68–15.7) for frequently in Gjakova ^e^	
						OR = 1.82 (0.30–11.1) for almost in Gjakova ^e^	
						OR = 0.68 (0.28–1.63) for seldom in Gjilan ^e^	
						OR = 1.41 (0.57–3.52) for frequently in Gjilan ^e^	
						OR = 1.02 (0.34–3.02) for almost in Gjilan ^e^	
						OR = 1.33 (0.66–2.71) for seldom in Peja ^e^	
						OR = 1.21 (0.56–2.60) for frequently in Peja ^e^	
						OR = 1.29 (0.54–3.10) for almost in Peja ^e^	
						OR = 0.81 (0.29–2.29) for seldom in Prishtina ^e^	
						OR = 1.57 (0.55–4.50) for frequently in Prishtina ^e^	
						OR = 0.73 (0.20–2.59) for almost in Prishtina ^e^	
						OR = 0.92 (0.50–1.69) for seldom in Prizren ^e^	
						OR = 0.88 (0.45–1.69) for frequently in Prizren ^e^	
						OR = 0.80 (0.33–1.96) for almost in Prizren ^e^	
Mphahlele et al., 2023 (South Africa) (72)	13–14	CS	3,957	Current asthma^a^	TRAP ^d^	OR = 1.423 (1.111–1.822) for truck frequency outside the respondent’s home *	Residence, fee- paying quintile, gender, body mass index, diet, exercise, television watching, sedentary computer use, pets, paracetamol>1/month in last 12 months, sibling, rhinoconjunctivitis, and eczema
Rathogwa-Takalani et al., 2024 (South Africa) (73)	13–14	CS	2,855	Ever asthma ^a^	TRAP ^d^	OR = 1.13 (0.74–1.73) for seldom through the day ^e^	Gender, community, born in study area, school, twin, type of fuel used in pets at home, currently smoking tobacco, smoking a water pipe, vigorous physical activity, use of paracetamol, and playing social games (social media and watching television)
						OR = 1.66 (0.99–2.00) for frequently through the day ^e^	
						OR = 1.64 (0.99–1.94) for almost whole day ^e^	
Faraji et al., 2024 (Iran) (74)	13–14	CS	1,118	Ever asthma ^a^	O_3_^c, 1^	OR = 0.86 (0.74–1.00) *	Gender, age, and smoking
					CO ^c, 1^	OR = 1.09 (0.54–2.27)	
					NO_2_^c, 1^	OR = 0.99 (0.97–1.01)	
					SO_2_^c, 1^	OR = 0.92 (0.81–1.05)	
					PM_10_^c, 1^	OR = 0.99 (0.98–1.00)	
					PM_2.5_^c, 1^	OR = 0.99 (0.96–1.02)	
				Current asthma^a^	O_3_^c, 1^	OR = 0.79 (0.70–0.89) *	
					CO ^c, 1^	OR = 1.02 (0.59–1.75)	
					NO_2_^c, 1^	OR = 0.99 (0.98–1.01)	
					SO_2_^c, 1^	OR = 0.96 (0.87–1.07)	
					PM_10_^c, 1^	OR = 0.99 (0.98–1.00)	
					PM_2.5_^c, 1^	OR = 1.00 (0.98–1.03)	
Zanobetti et al., 2024 (USA) (75)	11	CO	5,279	Ever asthma ^a^	NO_2_^c, 1^	OR = 1.23 (1.02–1.47) for first year of life exposure *	Mother’s education, parental asthma, smoking during pregnancy, child’s race and ethnicity, gender, neighborhood characteristics, and cohort
						OR = 1.30 (1.08–1.57) for mean of year 1–2 exposure *	
						OR = 1.31 (1.07–1.60) for mean of year 1–3 exposure *	
					PM_2.5_^c, 1^	OR = 1.18 (0.97–1.46) for first year of life exposure	
						OR = 1.25 (1.01–1.54) for mean of year 1–2 exposure *	
						OR = 1.30 (1.03–1.65) for mean of year 1–3 exposure *	
Jafarinodoshan et al., 2024 (Iran) (76)	13–14	CS	5,141	Ever asthma ^a^	TRAP ^d^	OR = 1.25 (0.98–1.59) for seldom through the day ^e^	Age, gender, race, type of house, and house floor
						OR = 1.89 (1.39–2.57) for frequently through the day ^e^	
						OR = 2.19 (1.33–3.62) for almost whole day ^e^	
Malamardi et al., 2024 (India) (77)	13–14	CS	3,051	Ever asthma ^a^	TRAP ^d^	OR = 1.638 (0.952–2.817) for passing of trucks near residence	Gender and body mass index
Qiu et al., 2024 (China) (78)	10–13	CS	4,146	Current asthma^a^	PM_2.5_^c,1^	OR = 1.252 (1.049–1.495)	Parental education level
Wang et al., 2025 (USA) (31)	10	CO	23,234	Current asthma^d^	PM_2.5_^c, 1^	HR = 1.19 (1.10–1.28)	Gender, race/ ethnicity, gestational smoking, maternal education, parental history of asthma, percent of low income, black, < high school education, unemployed, female householders, population density, decade of birth, and for site.
					NO_2_^c,1^	HR = 1.19 (1.05–1.34)	
					O_3_^c, 1^	HR = 1.11 (1.01–1.22)	

^a^ Parent- or child self-reported physician diagnosis; ^b^ Medical records; ^c^ Environmental measurement/modeling (e.g., fixed site monitor and land used regression); ^d^ Questionnaire or interview; ^e^ The frequency of buses/trucks pass by house, heavy truck traffic, density of road traffic, and residential proximity to major roadways (seldom, frequent, or constant); ^1^ Long-term exposure; ^2^ Short-term exposure; OR: odds ratio; HR: hazard ratio; RR: relative risk; PR: prevalence ratio; IRR: incidence rate ratio; POR: prevalence odds ratio; CS: cross-sectional study; CO: cohort study; PM_2.5_: particulate matter with diameter ≤ 2.5 micrometers; PM_10_: particulate matter with diameter ≤ 10 micrometers; CO: carbon monoxide; NO_2_: nitrogen dioxide; O_3_: ozone; SO_2_: sulfur dioxide; TRAP: traffic-related air pollution; μg/m*^3^*: microgram per cubic meter; ppm: parts per million; ppb: parts per billion; * *p* value <0.05.

### 3.3 Risk of bias

Quality assessment scores for the 51 included studies ranged from 5 to 11 out of a possible 14 points. Fourteen studies (27.5%) were rated as good (score = 11), thirty-three studies (64.7%) were rated as fair (scores 6–10), and four studies (7.8%) were rated as poor (score = 5). Studies classified as good typically demonstrated appropriate adjustment for key confounders and employed validated methods for exposure assessment. Common methodological limitations across the studies included the absence of sample size justification, inadequate exposure duration, reliance on self-reported exposure or outcome measures, use of single-timepoint exposure assessments, and limitations inherent to the study design. Full details of the quality appraisal are provided in Supplementary Table S2. The overall risk of bias was generally low, although concerns regarding external validity were common across studies.

### 3.4 Association between outdoor air pollution and adolescent asthma

Of the 51 studies included in the systematic review, 40 met the criteria for meta-analysis. Air pollutants were categorized into three groups, including particulate matters (PM_2.5_ and PM_10_), gaseous pollutants (CO, NO_2_, O_3_, and SO_2_), and TRAP. The pooled effect estimates were calculated for each.

#### 3.4.1 Particulate matters

**Figure 2:**
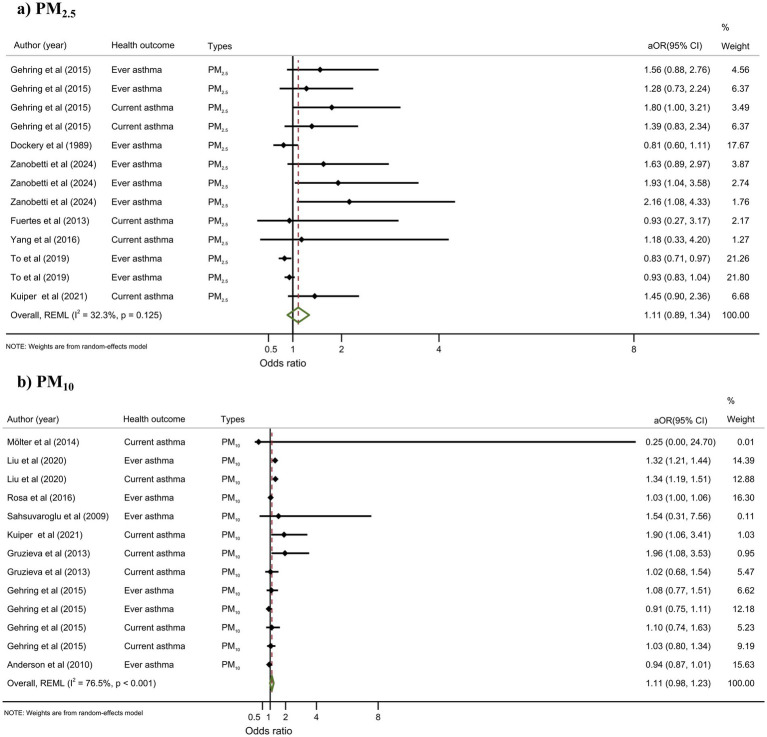
Meta-analysis of the association between particulate matters and adolescent asthma. **(a)** The association between PM2.5 and adolescent asthma; **(b)** The association between PM10 and adolescent asthma.

Eight studies assessed the association between PM_2.5_ and adolescent asthma. The reported effect sizes varied in direction; for example, To et al. (68) found a protective association (aOR = 0.82, 95% CI: 0.69–0.97), while Zanobetti et al. (75) reported an increased risk (aOR = 1.30, 95% CI: 1.03–1.65). The random-effects meta-analysis indicated a non-significant association between PM_2.5_ exposure and increased asthma risk (aOR = 1.11, 95% CI: 0.891.34) (Figure 2a). For PM₁₀, the meta-analysis included eight studies. Although some individual studies suggested a positive association, the overall random-effects estimate was not statistically significant (aOR = 1.11, 95% CI: 0.98–1.23) (Figure 2b).

#### 3.4.2 Gaseous pollutants

**Figure 3:**
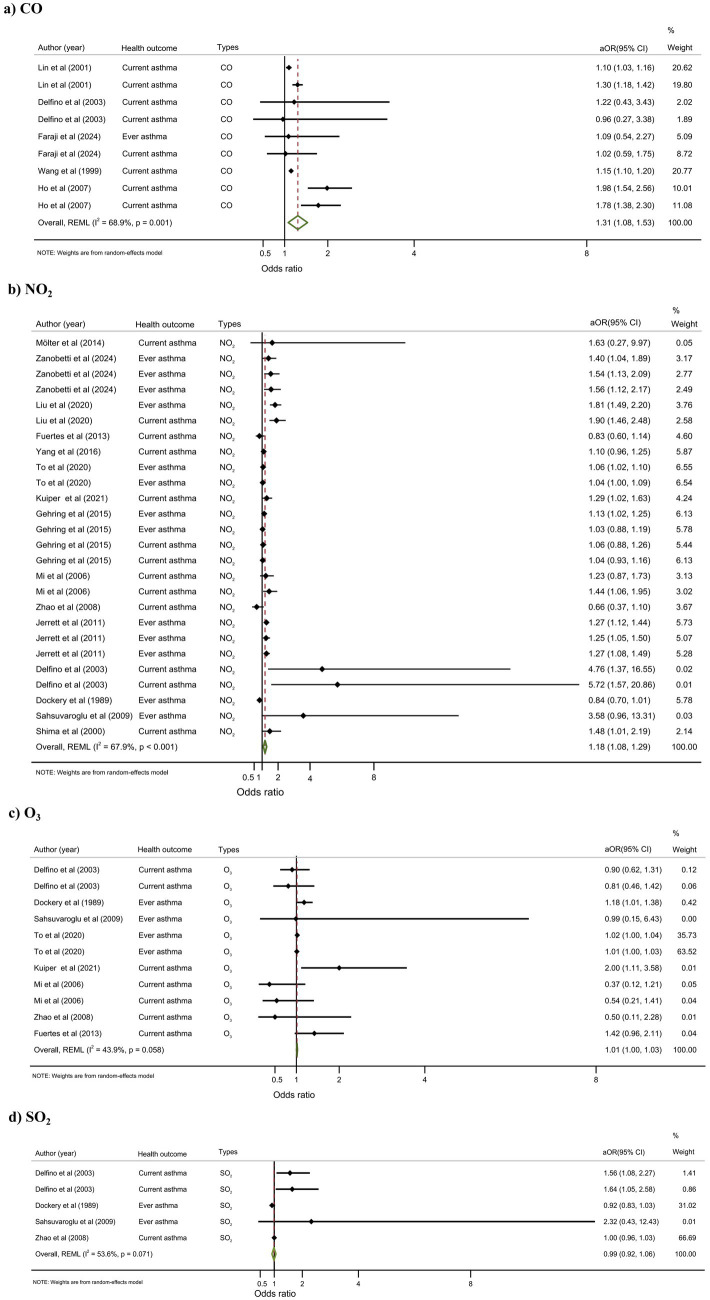
Meta-analysis of the association between gaseous pollutants and adolescent asthma. **(a)** The association between CO and adolescent asthma; **(b)** The association between NO_2_ and adolescent asthma; **(c)** The association between O_3_ and adolescent asthma; **(d)** The association between SO_2_ and adolescent asthma.

Five studies examined CO exposure. Despite variability in effect size, all studies showed a consistent direction of increased asthma risk. The pooled random-effects estimate confirmed CO as a significant risk factor (aOR = 1.31, 95% CI: 1.08–1.53) (Figure 3a). Fifteen studies investigated NO₂. Twelve studies reported a positive association (e.g., Kuiper et al. (70): aOR = 1.29, 95% CI: 1.02–1.63), while three reported a protective effect (e.g., Dockery et al. (36)). The random-effects meta-analysis showed a significant association between NO₂ exposure and asthma (aOR = 1.18, 95% CI: 1.08–1.29) (Figure 3b). Eight studies examined O₃. Five reported it as a risk factor (e.g., Dockery et al. (36): aOR = 1.90, 95% CI: 1.00–3.40), and three suggested a protective effect (e.g., Zhao et al. (46): aOR = 0.50, 95% CI: 0.11–2.28). The pooled analysis indicated a statistically significant but weak association between O₃ and asthma (aOR = 1.01, 95% CI: 1.00–1.03) (Figure 3c). For SO₂, the random-effects meta-analysis of four studies did not show a significant association with asthma (aOR = 0.99, 95% CI: 0.92–1.06) (Figure 3d).

#### 3.4.3 TRAP: eighteen studies assessed TRAP

**Figure 4:**
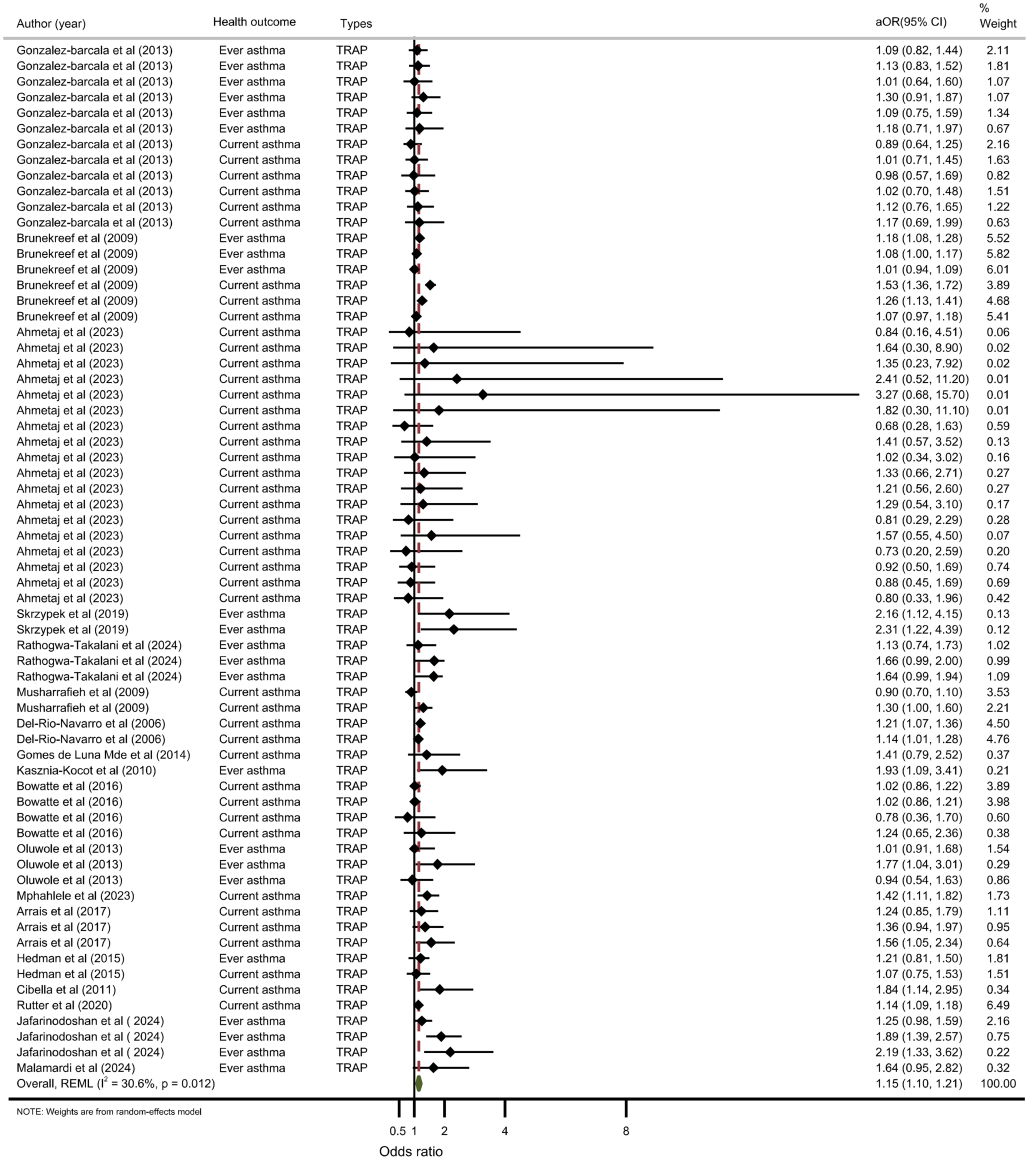
Meta-analysis of the association between TRAP and adolescent asthma.

While five studies suggested a protective effect (e.g., Gonzalez-Barcala et al. (16): aOR = 0.89, 95% CI: 0.64–1.25), the majority (*n* = 13) found an increased risk (e.g., Brunekreef et al. (47): aOR = 1.18, 95% CI: 1.08–1.28). The random-effects meta-analysis confirmed TRAP as a significant risk factor for adolescent asthma (aOR = 1.15, 95% CI: 1.10–1.21) (Figure 4).

### 3.5 Subgroup and sensitive analysis

Meta-analyses of seven outdoor air pollutants revealed varying degrees of heterogeneity. Sensitivity analyses were therefore conducted to explore potential sources of heterogeneity and assess the robustness of the pooled estimates. Results are summarized in Table 2.

**Table 2:** Subgroup and sensitivity analysis.

Air pollution	Type of air pollution	Condition for analysis		Studies included	Fixed-effect meta-analysis				Random-effect meta-analysis			
					aOR	95%CI	*I* ^2^	*p* value	aOR	95%CI	*I* ^2^	*p* value
Outdoor air pollution	PM_2.5_	All (Primary)	All (Cohort study only)	7	0.92	0.84–0.99	32.3%	0.125	1.11	0.89–1.34	32.3%	0.125

All (secondary)		All	8	1.02	0.95–1.09	77.3%	<0.001	1.27	1.04–1.51	77.3%	<0.001

Study design		Cohort study	7	0.92	0.84–0.99	32.3%	0.125	1.11	0.89–1.34	32.3%	0.125

Cross-sectional study		1	1.59	1.40–1.77	0.0%	0.919	1.59	1.40–1.77	0.0%	0.919

Outcome	Current asthma	5	1.54	1.28–1.80	0.0%	0.927	1.54	1.28–1.80	0.0%	0.927

Ever asthma	5	0.97	0.90–1.05	81.3%	<0.001	1.19	0.90–1.48	81.3%	<0.001

Exposure time	Short term exposure	0	NA	NA	NA	NA	NA	NA	NA	NA

Long term exposure	8	1.02	0.95–1.09	77.3%	<0.001	1.27	1.04–1.51	77.3%	<0.001

PM_10_	All	All	8	1.04	1.01–1.07	76.5%	<0.001	1.11	0.98–1.23	76.5%	<0.001

	Study design	Cohort study	4	1.00	0.88–1.13	0.0%	0.538	1.00	0.88–1.13	0.0%	0.538

	Cross-sectional study	4	1.04	1.02–1.07	91.0%	<0.001	1.15	0.95–1.35	91.0%	<0.001

	Outcome	Current asthma	5	1.24	1.12–1.37	22.8%	0.255	1.21	1.02–1.40	22.8%	0.255

Ever asthma	5	1.03	1.00–1.06	84.7%	<0.001	1.06	0.90–1.21	84.7%	<0.001

Exposure time	Short term exposure	1	1.03	1.00–1.06	NA	NA	1.03	1.00–1.06	NA	NA

Long term exposure	7	1.07	1.02–1.12	77.8%	<0.001	1.12	0.98–1.27	77.8%	<0.001

CO	All	All	5	1.16	1.12–1.19	68.9%	0.001	1.31	1.08–1.53	68.9%	0.001

	Study design	Cohort study	1	1.10	0.02–2.17	0.0%	0.814	1.10	0.02–2.17	0.0%	0.814

	Cross-sectional study	4	1.16	1.12–1.19	76.6%	<0.001	1.32	1.08–1.56	76.6%	<0.001

	Outcome	Current asthma	5	1.16	1.12–1.19	72.8%	<0.001	1.32	1.08–1.56	72.8%	<0.001

Ever asthma	1	1.09	0.23–1.96	NA	NA	1.09	0.23–1.96	NA	NA

Exposure time	Short term exposure	2	1.80	1.47–2.13	0.0%	0.532	1.80	1.47–2.13	0.0%	0.532

Long term exposure	3	1.15	1.11–1.19	52.8%	0.076	1.17	1.07–1.26	52.8%	0.076

NO_2_	All	All	15	1.07	1.05–1.10	67.9%	<0.001	1.18	1.08–1.29	67.9%	<0.001

	Study design	Cohort study	11	1.07	1.04–1.09	55.0%	0.002	1.11	1.04–1.18	55.0%	0.002

	Cross-sectional study	4	1.37	1.18–1.55	80.5%	<0.001	1.41	0.97–1.85	80.5%	<0.001

	Outcome	Current asthma	10	1.08	1.01–1.15	56.0%	0.007	1.15	0.97–1.33	56.0%	0.007

Ever asthma	7	1.07	1.04–1.10	76.2%	<0.001	1.21	1.07–1.34	76.2%	<0.001

Exposure time	Short term exposure	3	1.06	0.82–1.29	59.3%	0.043	1.12	0.65–1.58	59.3%	0.043

Long term exposure	12	1.07	1.05–1.10	70.6%	<0.001	1.19	1.08–1.29	70.6%	<0.001

O_3_	All	All	8	1.01	1.00–1.03	43.9%	0.058	1.01	1.00–1.03	43.9%	0.058

	Study design	Cohort study	5	1.01	1.00–1.03	34.7%	0.163	1.01	1.00–1.03	34.7%	0.163

	Cross-sectional study	3	0.46	0.09–0.84	0.0%	0.962	0.46	0.09–0.84	0.0%	0.962

	Outcome	Current asthma	5	0.85	0.64–1.06	48.4%	0.071	0.86	0.55–1.16	48.4%	0.071

Ever asthma	3	1.01	1.00–1.03	19.1%	0.295	1.01	1.00–1.03	19.1%	0.295

Exposure time	Short term exposure	3	0.72	0.50–0.95	0.0%	0.514	0.72	0.48–0.96	0.0%	0.514

Long term exposure	5	1.02	1.00–1.03	38.0%	0.153	1.02	1.00–1.03	38.0%	0.153

SO_2_	All	All	4	0.99	0.96–1.03	53.6%	0.071	0.99	0.92–1.06	53.6%	0.071

	Study design	Cohort study	2	0.95	0.85–1.05	73.4%	0.023	1.27	0.77–1.78	73.4%	0.023

	Cross-sectional study	2	1.00	0.97–1.04	0.0%	0.666	1.00	0.97–1.04	0.0%	0.666

	Outcome	Current asthma	2	1.00	0.97–1.04	67.0%	0.048	1.29	0.83–1.74	67.0%	0.048

Ever asthma	2	0.92	0.82–1.02	0.0%	0.647	0.92	0.82–1.02	0.0%	0.647

Exposure time	Short term exposure	2	1.00	0.97–1.04	67.0%	0.048	1.29	0.83–1.74	67.0%	0.048

Long term exposure	2	0.92	0.82–1.02	0.0%	0.647	0.92	0.82–1.02	0.0%	0.647

TRAP	All	All	18	1.13	1.10–1.15	30.6%	0.012	1.15	1.10–1.21	30.6%	0.012

	Study design	Cohort study	2	1.04	0.93–1.15	0.0%	0.871	1.04	0.93–1.15	0.0%	0.871

	Cross-sectional study	16	1.13	1.11–1.16	33.9%	0.007	1.17	1.11–1.23	33.9%	0.007

	Outcome	Current asthma	12	1.14	1.11–1.17	19.1%	0.141	1.14	1.06–1.21	19.1%	0.141

Ever asthma	9	1.10	1.06–1.15	45.2%	0.010	1.18	1.09–1.27	45.2%	0.010

Frequency of exposure	Seldom	7	1.04	0.98–1.09	0.0%	0.932	1.04	0.98–1.09	0.0%	0.932

	Frequent	9	1.13	1.07–1.19	37.3%	0.057	1.19	1.07–1.32	37.3%	0.057

Constant	10	1.26	1.18–1.34	25.0%	0.155	1.28	1.14–1.43	25.0%	0.155
			Unidentified	7	1.14	1.10–1.17	6.9%	0.377	1.14	1.10–1.17	6.9%	0.377

aOR, adjusted odds ratio; PM_2.5_, particulate matter with diameter ≤2.5 micrometers; PM_10_, particulate matter with diameter ≤10 micrometers; CO, carbon monoxide; NO_2_, nitrogen dioxide; O_3_, ozone; SO_2_, sulfur dioxide; TRAP, traffic-related air pollution; NA, not available.

#### 3.5.1 PM_2.5_ (8 studies)

Analysis of seven cohort studies using a random-effects model identified no statistically significant link between PM_2.5_ and adolescent asthma (aOR = 1.11, 95% CI: 0.89–1.34; *I*^2^ = 32.3%). Subgroup analyses showed that whole studies (*n* = 8) and cross-sectional study (*n* = 1) reported a strong association (aOR = 1.27, 95% CI: 1.04–1.51, *I*^2^ = 77.3%; aOR = 1.59, 95% CI: 1.40–1.77; *I*^2^ = 0.0%, respectively). Significant associations were found for current asthma (*n* = 5, aOR = 1.54, 95% CI: 1.28–1.80; *I*^2^ = 0.0%), but not for ever asthma. All studies assessed long-term exposure; no short-term exposure studies were available.

#### 3.5.2 PM₁₀ (8 studies)

The fixed-effects model indicated a marginally significant association (aOR = 1.04, 95% CI: 1.01–1.07), but heterogeneity was high (*I*^2^ = 76.5%). Under the random-effects model, the association was non-significant (aOR = 1.11, 95% CI: 0.98–1.23). Cross-sectional studies (*n* = 4) showed significance only under the fixed-effects model (aOR = 1.04, 95% CI: 1.02–1.07), with extreme heterogeneity (*I*^2^ = 91.0%). For current asthma (*n* = 5), associations were significant under both fixed-effects (aOR = 1.24) and random-effects (aOR = 1.21, *I*^2^ = 22.8%) models, but not for ever asthma. Long-term exposure (*n* = 7) showed borderline significance (random effects: aOR = 1.12), whereas the single short-term exposure study reported aOR = 1.03.

#### 3.5.3 CO (5 studies)

The pooled estimate under the random-effects model showed a significant association (aOR = 1.31, 95% CI: 1.08–1.53; *I*^2^ = 68.9%). Cross-sectional studies (*n* = 4) and current asthma (*n* = 5) showed consistent results (aOR = 1.32, *I*^2^ = 76.6 and 72.8%, respectively). No significant associations were observed in the cohort study or ever asthma. Notably, short-term exposure (aOR = 1.80, 95% CI: 1.47–2.13; *I*^2^ = 0.0%) had a stronger effect than long-term exposure (aOR = 1.17, *I*^2^ = 52.8%), suggesting acute responses.

#### 3.5.4 NO₂ (15 studies)

Pooled analysis demonstrated a significant positive association (aOR = 1.18, 95% CI: 1.08–1.29; *I*^2^ = 67.9%), corresponding to an 18% increase in asthma risk per 10 μg/m^3^ increment. Cohort studies (*n* = 11), ever asthma (*n* = 7), and long-term exposure (*n* = 12) all showed significant associations. Cross-sectional studies, current asthma, and short-term exposure groups did not show significant results.

#### 3.5.5 O₃ (8 studies)

Overall, the pooled estimate showed a marginal but significant association (aOR = 1.01, 95% CI: 1.00–1.03; *I*^2^ = 43.9%). Weak positive associations were found in cohort studies, ever asthma, and long-term exposure groups. In contrast, cross-sectional studies and short-term exposure analyses reported protective effects (e.g., aOR = 0.46, 95% CI: 0.09–0.84), possibly due to reverse causality or behavioral confounding (e.g., staying indoors during high O₃ levels). Current asthma studies showed no significant effect (aOR = 0.86, *I*^2^ = 48.4%).

#### 3.5.6 SO₂ (4 studies)

No overall association was found (aOR = 0.99, 95% CI: 0.92–1.06; *I*^2^ = 53.6%). Cohort studies suggested elevated risk (aOR = 1.27, *I*^2^ = 73.4%), while cross-sectional studies showed no association (aOR = 1.00, *I*^2^ = 0.0%). Subgroup analyses for current asthma and short-term exposure suggested increased risk (aOR = 1.29, *I*^2^ = 67.0%), though estimates were imprecise.

#### 3.5.7 TRAP (18 studies)

The pooled effect size confirmed a significant association between TRAP and asthma (fixed-effects aOR = 1.13; random-effects aOR = 1.15, *I*^2^ = 30.6%). Cross-sectional studies (*n* = 16) yielded a stronger association (aOR = 1.17) than cohort studies (*n* = 2, aOR = 1.04). Both current and ever asthma outcomes showed significant associations. Importantly, a dose–response relationship was evident: seldom exposure showed no significant effect, frequent exposure increased risk by 13–19%, and constant exposure was associated with the strongest effect (aOR = 1.26, 95% CI: 1.18–1.34; *I*^2^ = 25.0%). Heterogeneity across these strata was low to moderate (*I*^2^ = 0–45.2%).

### 3.6 Publication bias and funnel plot

**Figure 5:**
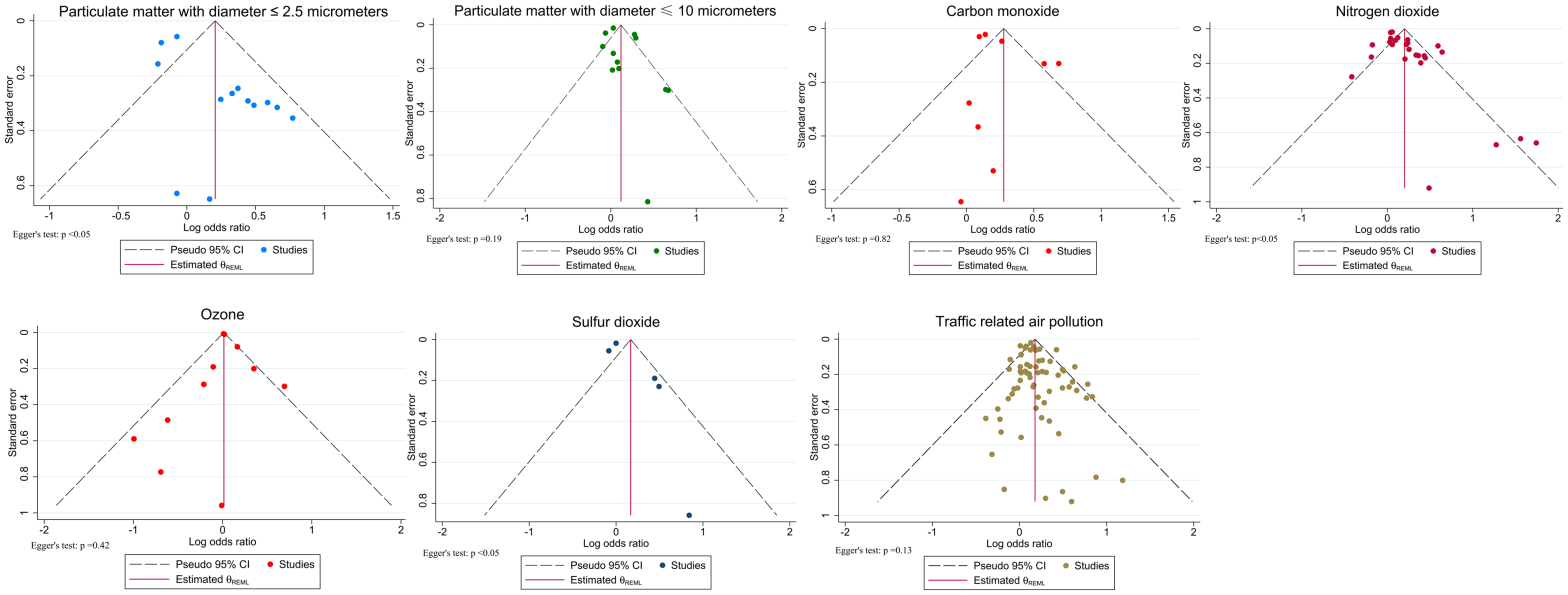
Funnel plots.

Publication bias was evaluated using Egger’s regression test and visual inspection of funnel plots. Statistically significant asymmetry (Egger’s test *p* value < 0.05) was detected for PM_2.5_, NO₂, and SO₂, suggesting potential publication bias. Funnel plots for these pollutants revealed skewed distributions, with smaller studies tending to report larger effect sizes. For all other pollutants, Egger’s test results were non-significant (*p* value > 0.05), and corresponding funnel plots showed no notable asymmetry (Figure 5).

To further assess and adjust for potential bias, nonparametric trim-and-fill analysis was conducted. For NO₂, two studies were imputed under the random-effects model, slightly reducing the pooled estimate while maintaining statistical significance (OR = 1.21, 95% CI: 1.10–1.33; original OR = 1.22, 95% CI: 1.11–1.34). The SO₂ analysis imputed two studies, which also reduced the association but still non-significant (OR = 1.08, 95% CI: 0.83–1.40; original OR = 1.18, 95% CI: 0.90–1.56), indicating the observed associations for NO₂ and SO₂ are robust to corrections for potential publication bias. However, for PM_2.5_, eight studies were implemented under the random-effects model, the pooled effect estimate was substantially attenuated from a borderline significant to non-significant (OR = 0.89, 95% CI: 0.70–1.12; original OR = 1.23, 95% CI: 1.00–1.51), this indicates that, after accounting for publication bias, the initial positive association was no longer significant, and the results support no significant association between PM_2.5_ and adolescent asthma.

## 4 Discussion

To our knowledge, this is the systematic review and meta-analysis to comprehensively synthesize epidemiological evidence on the association between outdoor air pollution and asthma in adolescents. Quantitative analyses of seven major combustion-related pollutants revealed significant positive associations between exposure to CO, NO₂, O₃, and TRAP and increased asthma risk. In contrast, PM_2.5_, PM₁₀ and SO₂ were not significantly associated with asthma in the pooled estimates.

For PM_2.5_, a primary meta-analysis restricted to cohort studies (*n* = 7) was conducted to strengthen causal inference. The pooled estimate was not statistically significant (aOR = 1.11; 95% CI: 0.89–1.34; *I*^2^ = 32.3%). Crucially, this null association remained non-significant, and the effect estimate was often attenuated, following adjustment for substantial publication bias. Accordingly, the most methodologically robust evidence within our review does not support a significant association between long-term PM_2.5_ exposure and adolescent asthma. Kamarehei et al. (79), using the AirQ^+^ model in Poldokhtar, Iran, provided a mechanistic and methodological explanation for this finding, showing that the health effects of long-term PM_2.5_ exposure vary substantially by concentration, emission source, and health outcome. Regional heterogeneity in PM_2.5_ composition contributes to divergent toxicological profiles, with rural or natural-source PM_2.5_ often differing markedly from urban anthropogenic particles (80–83), potentially reducing its ability to induce allergic Th2 immune responses (84, 85). The characteristics and outcomes of the seven included cohort studies further support this interpretation. Moreover, the attributable burden of PM_2.5_ is predominantly observed in adult chronic diseases such as chronic obstructive pulmonary disease, ischemic heart disease, and lung cancer, while adolescent asthma has not shown a statistically significant association (86). As a multifactorial disease, its weaker signal may be masked by the dominant burden of adult morbidity. Additionally, short-term PM_2.5_ exposure appears to exert a stronger effect on respiratory morbidity than long-term exposure (86). In summary, this meta-analysis found no significant association between long-term PM_2.5_ exposure and adolescent asthma, and this null finding persisted after adjustment for publication bias. Clarifying this relationship will require large, preregistered prospective cohorts with result-independent publication.

Interestingly, in the secondary analysis, PM_2.5_ exposure was significantly associated with adolescent asthma (aOR = 1.27; 95% CI: 1.04–1.51) with significant heterogeneity (*I*^2^ = 77.3%) arose from multiple methodological sources with significant heterogeneity (*I*^2^ = 77.3%) arose from multiple methodological sources. First, study design contributed to bias, while one cross-sectional study reported strong associations (aOR = 1.59, 95% CI: 1.40–1.77), cohort studies (*n* = 7) showed null effects (aOR = 1.11, 95% CI: 0.89–1.34), likely due to reverse causation and residual confounding. Adjustments varied across studies. Some studies accounted for indoor pollutants (55), others adjusted only for smoking (74), affecting effect size estimates. Second, inconsistent asthma definitions introduced misclassification: studies using current asthma showed stronger associations (aOR = 1.54), possibly due to symptom-based overreporting, whereas ever asthma definitions may underestimate risk by including remitted cases and relying on incomplete records. Third, exposure misclassification likely resulted from variation in PM_2.5_ assessment methods, e.g., gridded models (75) vs. LUR models (67), with differing spatial and temporal resolutions, potentially biasing associations toward the null, as seen in Dockery et al. (36) and To et al. (68). Fourth, only three studies explicitly assessed early-life exposure across multiple time points (58, 68, 75); others lacked clear exposure windows. Fifth, limited eligible studies constrained analysis. Of 12 identified, three were excluded due to unspecified increments (27, 28, 31, 74), leaving eight. The absence of short-term exposure data and unadjusted co-pollutants (e.g., NO₂, O₃) further obscured dose–response patterns. Additional variability likely stems from unmeasured modifiers such as age (11 years in Zanobetti et al. (75) vs. 17 years in To et al. (68)) and regional differences in PM_2.5_ composition.

A critical finding from our meta-analysis of PM₁₀ warrants cautious interpretation. Although the pooled estimate was not statistically significant, the high heterogeneity (*I*^2^ = 76.5%) indicates substantial inconsistency across studies rather than uniform evidence of no effect. Thus, it would be premature to conclude the absence of an association between PM₁₀ and asthma; instead, the evidence remains inconclusive and context-dependent. The non-significant pooled estimate for long-term PM₁₀ exposure and adolescent asthma likely reflects multiple, overlapping factors. First, PM₁₀ composition varies widely across regions (e.g., crustal dust vs. anthropogenic emissions), resulting in differential toxicity (87). Second, adolescents may exhibit distinct exposure patterns or physiological resilience compared with adults or younger children (88). Third, long-term exposure tends to produce weaker health effects than short-term exposure (89). Finally, methodological differences, such as exposure assessment precision and adjustment for confounders like indoor allergens or socioeconomic status, may have attenuated the observed associations (90, 91).

Of the 13 eligible studies, five did not report exposure increments and were excluded to ensure comparability and data integrity (37, 40, 45, 66, 74). The remaining eight studies were included in the analysis. Notably, substantial heterogeneity was observed, particularly among cross-sectional designs and studies examining ever asthma or long-term exposure. This variability may reflect methodological inconsistencies and differences in population characteristics. Substantial heterogeneity in the PM₁₀–asthma association largely reflects differences in study design. Cohort studies (*I*^2^ = 0.0%) reported no significant association, contrasts sharply with the significant positive association in the cross-sectional study (*I*^2^ = 91.0% despite very high heterogeneity), likely influenced by reverse causation and recall bias. Although cohort designs better infer temporality, their small sample sizes (*n* = 4) and exposure misclassification from residential mobility may obscure associations. Outcome definitions also contributed: current asthma analyses showed more consistent associations (*I*^2^ = 22.8%) compared to ever asthma (*I*^2^ = 84.7%), reflecting diagnostic variability and recall differences. Exposure assessment methods varied widely—e.g., personal monitors (60) vs. fixed-site data (67)—along with differences in temporal windows and co-pollutant adjustment. Gruzieva et al. (53) found early-life exposure associated with higher asthma risk (aOR = 1.96), unlike later exposure (aOR = 1.02), highlighting critical developmental windows. Gehring et al. (58) reported location-based differences (birth vs. current address), illustrating exposure misclassification from migration. Population-level modifiers also shaped associations: Sahsuvaroglu et al. (49) found null effects in girls without hay fever, whereas Kuiper et al. (70) observed elevated risk in other subgroups (aOR = 1.90), pointing to gene–environment interactions and host susceptibility. Thus, the non-significant pooled estimate for PM₁₀ should be viewed not as evidence of absence, but as evidence of inconsistency across studies. Future research employing rigorous longitudinal designs with precise exposure assessment is needed to resolve these discrepancies and clarify the true association.

Meta-analysis revealed that exposure to CO was significantly associated with adolescent asthma (aOR = 1.31, 95% CI: 1.08–1.53). CO contributes to the onset and exacerbation of adolescent asthma through three interrelated mechanisms. CO’s high binding affinity to hemoglobin induces systemic hypoxia (92), which promotes bronchial smooth muscle contraction and activates hypoxia-inducible factors (93), intensifying airway inflammation characterized by increased mucus secretion, mucosal edema, and immune cell infiltration (94). CO also disrupts redox homeostasis by elevating reactive oxygen species (ROS) and impairing antioxidant defenses (95, 96), thereby initiating oxidative stress and damaging airway epithelial integrity. This, in turn, activates proinflammatory pathways such as nuclear factor-kappa B (NF-κB), amplifying asthma-related inflammation (97). Lastly, the combined effect of hypoxia, oxidative stress, and inflammation lowers airway reactivity thresholds, enhancing susceptibility to environmental triggers and worsening symptom severity in adolescents (98). Substantial heterogeneity in the CO meta-analysis (*I*^2^ = 68.9%) arose from both methodological and biological variability. Study design played a major role. Cross-sectional studies (*n* = 4; *I*^2^ = 76.6%) reported higher effect estimates (aOR = 1.32), likely due to temporal ambiguity and incomplete confounder control, such as partial adjustment for smoking and unaccounted co-pollutants (37, 74). In contrast, the null finding in underpowered single cohort study (aOR = 1.10, 95% CI: 0.02–2.17) likely reflects insufficient power. Exposure assessment varied widely: annual means, 8-h means, geographic information system-based estimates, and hourly maxima with inconsistent increments (37, 39, 41, 45, 74). Despite unit harmonization efforts, some metrics remained non-convertible, though excluding Delfino et al. (41) had minimal impact on pooled estimates (aOR = 1.32 vs. 1.31). Long-term exposures were vulnerable to spatial misclassification and seasonal variation. Short-term exposure showed stronger associations (aOR = 1.80, *I*^2^ = 0.0%) than long-term (aOR = 1.17, *I*^2^ = 52.8%), likely due to acute hypoxic effects and latency bias. Clinical heterogeneity persisted due to inconsistent asthma definitions (self-report vs. diagnosis), limited stratification by modifiers (e.g., gender in Ho et al. (45)), and lack of endotype-specific analyses. Data on ever asthma was limited to a single recall-based study.

The meta-analysis identified NO₂ as a significant risk factor for adolescent asthma (aOR = 1.18; 95% CI: 1.08–1.29). The persistence of a statistically significant association after this adjustment reinforces the robustness of the finding and reduces the likelihood that it reflects a publication bias effect. NO₂ exacerbates asthma through multiple synergistic pathways. Upon inhalation, it dissolves in airway mucosa, generating reactive nitrogen species and ROS (99), depleting antioxidants like glutathione, inducing oxidative stress, disrupting epithelial barriers, and enhancing allergen penetration (100). It also activates NF-κB and upregulates interleukin-33 (IL-33) and thymic stromal lymphopoietin (TSLP) (101), promoting dendritic cell-driven Th2 polarization (increased interleukin-4 (IL-4), interleukin-5 (IL-5), interleukin-13 (IL-13)) (102), leading to eosinophilic inflammation, immunoglobulin E (IgE) production, and mucus hypersecretion (103). Concurrently, oxidative stress upregulates transient receptor potential vanilloid 1 (TRPV1) and transient receptor potential ankyrin 1 (TRPA1) channels (104), triggering neurogenic inflammation and airway hyperresponsiveness (105). Long-term exposure alters immune gene expressions (e.g., forkhead box protein P3, IL-4) via DNA methylation, impairing regulatory T cells (Treg) function and contributing to airway remodeling and asthma persistence (106). However, the findings should be interpreted with caution due to substantial heterogeneity (*I*^2^ = 67.9%). Substantial heterogeneity in the NO₂ meta-analysis stems from multiple methodological sources. First, exposure measurement inconsistencies: of 23 eligible studies, 8 were excluded for lacking standardized effect estimates or using incompatible units (e.g., μg/m^3^, ppm, ppb). The remaining 15 studies were standardized to 10 μg/m^3^, but conversion assumptions (1 atm, 25 °C) may not reflect regional conditions, introducing systematic error. Second, population variability contributed, as studies spanned ages 10–18 with wide age ranges and differing sex distributions (e.g., Kuiper et al. (70); Liu et al. (67)). Third, study design influenced heterogeneity: while both cohort and cross-sectional studies showed positive associations, the latter may inflate estimates due to temporal ambiguity, while the former may retain residual confounding. Fourth, sample size varied widely (*n* = 19–22,574), with smaller studies often reporting larger, less precise effects (e.g., Delfino et al. (41)). Although trim-and-fill analysis showed minimal publication bias impact, imbalance in study size remained influential. Fifth, exposure assessment methods differed: studies used LUR models, satellite-integrated predictions, and ground monitoring with varying spatial resolutions, leading to classification inconsistencies. Sixth, asthma definitions varied, with higher heterogeneity in ever asthma likely due to recall bias. Seventh, exposure duration influenced findings—long-term exposure was generally positively associated with asthma (e.g., Yang et al. (61)), whereas short-term exposure findings were mixed (e.g., Zhao et al. (46)). Lastly, confounder adjustment strategies differed, ranging from socioeconomic status (62) to indoor pollution control (68), further contributing to inter-study variability.

O₃ exposure is associated with increased adolescent asthma risk (aOR = 1.01, 95% CI: 1.00–1.03; *I*^2^ = 43.9%). Its pathogenesis centers on potent oxidative activity that compromises airway epithelial integrity. O₃ reacts with alveolar surface lipids to generate ROS (107), depletes antioxidants like glutathione, and disrupts tight junction proteins (e.g., e-cadherin, zonula occluden-1), impairing epithelial barrier function (108, 109). This epithelial injury triggers the release of alarmins IL-33 and TSLP (110), activates innate lymphoid cell types 2 (ILC2) and T cells, and promotes IL-5/IL-13-driven eosinophilic and interleukin-17A-mediated neutrophilic inflammation—resulting in mixed airway inflammation (111, 112). O₃ also disrupts adaptive immunity and reduces treatment efficacy by enhancing Th2/ T helper type 17 cell (Th17) polarization, impairing Treg function, and increasing glucocorticoid receptor β expression while inactivating surfactant protein D through oxidative stress (113–116). These mechanisms may explain observed associations between long-term O₃ exposure and impaired small airway function (117). However, the primary source of heterogeneity in the O₃ meta-analysis was study design. Cohort studies (*I*^2^ = 34.7%, aOR = 1.01) showed a weak positive association, while cross-sectional studies (*I*^2^ = 0.0%, aOR = 0.46) indicated an inverse relationship, likely reflecting methodological differences. Exposure duration also contributed: long-term analyses (*I*^2^ = 38.0%, aOR = 1.02) supported a chronic risk, possibly linked to oxidative stress, whereas short-term studies (*I*^2^ = 0.0%, aOR = 0.72) may reflect seasonal confounding or insufficient acute exposure data. Outcome definitions influenced estimates, with current asthma (*I*^2^ = 48.4%, aOR = 0.85) showing weaker associations than ever asthma (*I*^2^ = 19.1%, aOR = 1.01), likely due to recall bias and case misclassification. Most studies relied on self-reported diagnoses, limiting validity. Exposure assessment varied—ranging from ultraviolet photometry and passive diffusion to LUR models (41, 43, 55), introducing spatial and temporal misclassification, particularly relevant for O₃ with smaller effect sizes. Only 8 of 13 studies were included in meta-analysis due to inconsistent or unconvertible exposure units. Despite efforts to harmonize increments (e.g., ppb to μg/m^3^ using standard conditions), regional variability likely introduced additional error. Overall, the observed heterogeneity stems largely from methodological variation rather than true biological differences.

Of the 13 studies on SO₂ and adolescent asthma, only 4 were included in the meta-analysis due to unclear exposure increments in the remaining 9 (e.g., Chiang et al. (15); Faraji et al. (74); Radhakrishnan et al. (27)). The pooled estimate for SO_2_ was not statistically significant, but this finding is characterized by high heterogeneity, sensitivity analysis confirmed the robustness of the SO₂ association after adjusting for publication bias. Substantial heterogeneity was observed (*I*^2^ = 53.6%), with greater variability in cohort (*I*^2^ = 73.4%), current asthma (*I*^2^ = 67.0%), and short-term exposure (*I*^2^ = 67.0%) subgroups. Contributing factors include: (1) small subgroup sizes (*n* = 2), amplifying random error; (2) inconsistencies in unit conversion, most studies used ppb, converted using a factor of 2.619 under standard conditions, though regional climate and pollution differences (e.g., US, Canada, China) likely introduced error; (3) contradictory effect directions (e.g., Dockery et al. (36): protective vs. Delfino et al. (41): harmful); (4) methodological heterogeneity, cohort studies (aOR = 1.27) used dynamic monitoring, while cross-sectional studies (aOR = 1.00) relied on retrospective data prone to recall bias; (5) variation in exposure duration, Delfino et al. (41) found 1-h peaks associated with increased asthma risk (aOR = 2.36), while longer exposures showed attenuated effects (aOR = 1.91 for 8-h max); (6) effect modification, Sahsuvaroglu et al. (49) reported stronger associations in girls without hay fever, while Zhao et al. (46) found null results in unstratified analyses. These findings suggest that observed heterogeneity reflects an interplay between exposure timing and host immune status. Acute SO₂ effects in sensitized individuals may be genuine, while inverse associations may reflect survivor bias (e.g., severe asthmatics relocating from polluted areas).

The meta-analysis indicated that TRAP significantly increases adolescent asthma risk (aOR = 1.15, 95% CI: 1.10–1.21). Mechanistically, inhaled TRAP components—such as diesel particles, NO₂, and O_3_, disrupt the respiratory epithelial barrier by degrading tight junction proteins (e.g., occludin, claudin-5), enhancing allergen penetration (118, 119). Concurrently, ROS depleted antioxidant defenses, a vulnerability heightened in adolescents due to an underdeveloped nuclear factor erythroid 2-related factor 2 pathway (120, 121). Epithelial injury triggers release of alarmins (TSLP, IL-33), activating ILC2 and initiating type 2 innate inflammation (122, 123). Polycyclic aromatic hydrocarbons (PAHs) further impair Treg function via the aryl hydrocarbon receptor, promoting Th2/Th17 polarization (124). Neuro-immune crosstalk amplifies damage through TRAP-induced activation of TRPV1/TRPA1 channels, releasing neuropeptides and triggering neurogenic inflammation that interacts with ILC2 to drive bronchial hyperresponsiveness and airway remodeling (125, 126).

This systematic review and meta-analysis synthesize robust epidemiological evidence linking outdoor air pollution to increased adolescent asthma risk, though key limitations remain. Most studies focused on NO₂ and TRAP, with limited data on PM_2.5_, PM₁₀, CO, O₃, and SO₂, warranting cautious interpretation for these pollutants. A key limitation of this meta-analysis is the widespread reliance on fixed-site ambient monitoring data for individual exposure assessment. This method does not capture individual mobility patterns or time spent indoors, leading to non-differential exposure misclassification. Because such errors are typically unrelated to health outcomes, it tends to bias effect estimates toward the null (127). Therefore, the pooled risk estimates in this analysis likely underestimate the true association between ambient air pollution and adolescent asthma. If exposure were measured with greater individual precision, the true effect sizes would likely be stronger. This limitation has important policy implications, suggesting that the actual public health burden of air pollution may exceed current estimates and that reducing exposure could yield even greater respiratory health benefits for adolescents. Asthma definitions also varied (self-report vs. clinical diagnosis), contributing to endpoint heterogeneity. Despite a broad search strategy, non-English or unpublished studies may have been missed, introducing potential language and publication bias (128, 129). However, funnel plots and Egger tests indicated minimal bias for most pollutants, and trim-and-fill analysis confirmed the robustness of PM_2.5_, NO₂ and SO₂ effect estimates. Many studies lacked adjustment for key confounders (e.g., allergy history, socioeconomic status) and varied in exposure metrics and time windows, affecting estimate validity. Predominantly cross-sectional designs further limit causal inference (130). High heterogeneity was evident in several subgroups (e.g., PM_2.5_, PM₁₀, CO, NO₂), likely driven by differences in study design, population levels, and analytical methods. Additional gaps include limited analysis of developmental stage–specific effects, underrepresentation of low-income or genetically vulnerable populations, and lack of data on secondary pollutants (e.g., O_3_-terpene–derived formaldehyde). Moreover, subgroup analyses by finer age, region, or socioeconomic factors were not feasible due to data limitations.

To address existing methodological limitations and refine causal inference, future research should adopt synergistic strategies. Emphasis should be placed on life-course exposure modeling within well-designed cohort studies to capture temporal variations and developmental susceptibility. Standardization of asthma diagnosis is essential and should involve a composite reference incorporating spirometry, validated biomarkers (e.g., fractional exhaled nitric oxide), and harmonized questionnaires, particularly adapted for use in resource-constrained settings. Moreover, accurate differentiation between long- and short-term exposure windows requires the application of exposure assessment models with high spatiotemporal resolution to minimize misclassification bias. Finally, future analyses should incorporate stratification by co-pollutant exposures, individual susceptibility factors, and epigenetic profiles, to better elucidate effect modification and improve the precision of risk estimates.

The interpretation of our findings should consider the methodological quality of the included studies. Common risks of bias in study design, exposure assessment, and confounder control may limit generalizability, though the overall low risk in analytical domains supports the internal validity of the pooled estimates. Our results, highlighting a significant association between outdoor air pollutants and adolescent asthma, must be viewed within global and national public health contexts. The WHO’s 2020 physical activity guidelines recommend that adolescents engage in at least 60 min of moderate-to-vigorous activity daily (131), a key strategy in combating non-communicable diseases (NCD), also prioritized in Iran’s national NCD prevention plan. However, in areas with poor air quality, encouraging outdoor activity may inadvertently increase exposure to asthma-inducing pollutants, posing a dilemma for adolescents, especially those with pre-existing asthma. Achieving global health targets thus requires integrated approaches that address both physical inactivity and air pollution. Effective interventions may include air quality alert systems to guide outdoor activity scheduling, development of urban “clean air zones” and green spaces, and provision of indoor exercise facilities during high-pollution episodes. Aligning environmental health and NCD prevention policies can foster synergistic strategies that protect respiratory health while promoting overall adolescent wellbeing.

## 5 Conclusion

This systematic review and meta-analysis provide evidence that exposure to specific outdoor air pollutants, including CO, NO₂, O₃, and TRAP, is significantly associated with increased risk of adolescent asthma. Among these, NO_2_ and CO showed the strongest associations, underscoring their critical role in adolescent respiratory pathogenesis. However, the interpretation of pooled estimates must be approached with caution due to notable heterogeneity across studies, arising from methodological disparities in exposure assessment, asthma definition, study design, and population characteristics. While publication bias and residual confounding may influence effect estimates, sensitivity and trim-and-fill analyses affirmed the robustness of the core findings.

These results underscore the urgent need for refined epidemiological approaches and international collaboration to advance exposure science, harmonize diagnostic criteria, and identify vulnerable subpopulations. Without such improvements, current risk estimates likely underestimate the true pulmonary burden of ambient air pollution on adolescents. Public health policies aimed at reducing air pollution exposure remain critical to mitigating the rising burden of asthma and promoting respiratory health across the course of life.

## Data Availability

The original contributions presented in the study are included in the article/supplementary material, further inquiries can be directed to the corresponding author.

## Glossary

WHO: World Health Organization
95% CI: 95% Confidence interval
PM_2.5_: Particulate matter with diameter ≤2.5 micrometers
NO₂: Nitrogen dioxide
O₃: Ozone
Th1: T helper type 1 cell
Th2: T helper type 2 cell
aOR: Adjusted odds ratio
μg/m^3^: Microgram per cubic meter
ppm: Parts per million
ppb: Parts per billion
SR: The systematic review
PRISMA-P: Preferred Reporting Items for Systematic Review and Meta-Analysis Protocols
MOOSE: Meta-analysis of Observational Studies in Epidemiology
Mesh: Medical Subjects Heading
OR: Odds ratio
HR: Hazard ratio
RR: Relative risk
PR: Prevalence ratio
IRR: Incidence rate ratio
POR: Prevalence odds ratio
CO: Carbon monoxide
PM_10_: Particulate matter with diameter ≤ 10 micrometers
SO_2_: Sulfur dioxide
TRAP: Traffic-related air pollution
LUR: Land use regression
NHLBI: National Heart, Lung, and Blood Institute
βeta: Regression coefficients
SE: Standard errors
ROS: Reactive oxygen species
NF-κB: Nuclear factor-kappa B
IL-33: Interleukin-33
TSLP: Thymic stromal lymphopoietin
IL-4: Interleukin-4
IL-5: Interleukin-5
IL-13: Interleukin-13
IgE: Immunoglobulin E
TRPV1: Transient receptor potential vanilloid 1
TRPA1: Transient receptor potential ankyrin 1
Treg: Regulatory T cells
ILC2: Innate lymphoid cell types 2
Th17: T helper type 17 cell
PAHs: Polycyclic aromatic hydrocarbons
NCD: Non-communicable diseases
*: *p* value < 0.05
CS: Cross-sectional study
CO: Cohort study
CC: Case–control study
