# Supplementary material for: Outdoor air pollutants and asthma risk in adolescents: evidence from a systematic review and meta-analysis
Source: Front Public Health. 2025 Dec 10;13:1721233. doi: 10.3389/fpubh.2025.1721233 (PMC12727952; doi:10.3389/fpubh.2025.1721233)
Supplement: Supplementary file 1 [file Table_1.docx]

**Supplementary Table S1** **The original effect measure, the incidence/prevalence value (s) used for conversion, and the final converted OR**

| **Author (year)** | **HO** | **Types** | **Original effect measure OR/POR/HR (95%CI)** | **Exposure unit** | **Exposure increment** | **Incidence or prevalence value****(s)** | **Final converted OR** | **Included in meta–**  **analysis** |
| --- | --- | --- | --- | --- | --- | --- | --- | --- |
| Dockery et al (1989) | EA | PM_2.5_ | OR=0.60 (0.30–1.40) | μg/m³ | 24.9μg/m³ | NA | OR=0.81 (0.60–1.11) | Yes |
| Islam et al (2007) | EA | PM_2.5_ | HR=0.65 (0.41–1.03) for low PM_2.5_>10^th^–90^th^ PR FVC | μg/m³ | NR | 0.64% | HR=0.65 (0.41–1.03) | No |
|  |  |  | HR=0.46 (0.30–0.71) for low PM_2.5_>10^th^–90^th^ PR FEV_1_ * |  |  |  | HR=0.46 (0.30–0.71) |  |
|  |  |  | HR=0.34 (0.21–0.56) for low PM_2.5_ >10^th^–90^th^ PR FEF_25–75_ * |  |  |  | HR=0.34 (0.21–0.56) |  |
|  |  |  | HR=1.41 (0.87–2.26) for high PM_2.5_>10^th^–90^th^ PR FVC |  |  | 1.59% | HR=1.41 (0.87–2.26) |  |
|  |  |  | HR=1.08 (0.66–1.76) for high PM_2.5_>10^th^–90^th^ PR FEV_1_ |  |  |  | HR=1.08 (0.66–1.76) |  |
|  |  |  | HR=0.76 (0.45–1.26) for high PM_2.5_>10^th^–90^th^ PR FEF_25–75_ |  |  |  | HR=0.76 (0.45–1.26) |  |
| Fuertes et al (2013) | CA | PM_2.5_ | OR=0.97 (0.59–1.58) | μg/m³ | 4 μg/m³ | NA | OR=0.93 (0.27–3.17) | Yes |
| Gehring et al (2015) | EA | PM_2.5_ | OR=1.25 (0.94–1.66) for road traffic at the birth address | μg/m³ | 5 µg/m³ | NA | OR=1.56 (0.88–2.76) | Yes |
|  |  |  | OR=1.13 (0.85–1.49) for road traffic at the current address |  |  |  | OR=1.28 (0.73–2.24) |  |
|  | CA | PM_2.5_ | OR=1.34 (1.00–1.79) for road traffic at the birth address * |  |  |  | OR=1.80 (1.00–3.21) |  |
|  |  |  | OR=1.18 (0.91–1.53) for road traffic at the current address |  |  |  | OR=1.39 (0.83–2.34) |  |
| Yang et al (2016) | CA | PM_2.5_ | OR=1.02 (0.87–1.18) | μg/m³ | 1.2 µg/m³ | NA | OR=1.18 (0.33–4.20) | Yes |
| Liu et al (2020) | EA | PM_2.5_ | OR=1.70 (1.45–2.01) * | μg/m³ | 11.53μg/m³ | NA | OR=1.58 (1.38–1.83) | Yes |
|  | CA | PM_2.5_ | OR=1.72 (1.37–2.15) * |  |  |  | OR=1.60 (1.32–1.95) |  |
| To et al (2020) | EA | PM_2.5_ | OR=0.82 (0.69–0.97) for at birth exposure | μg/m³ | 10.63µg/m³ | NA | OR=0.83 (0.71–0.97) | Yes |
|  |  |  | OR=0.92 (0.81–1.04) for at 3-year exposure |  | 11µg/m³ |  | OR=0.93 (0.83–1.04) |  |
| Kuiper et al (2021) | CA | PM_2.5_ | OR=1.45 (0.90–2.36) | μg/m³ | 10 µg/m³ | NA | OR=1.45 (0.90–2.36) | Yes |
| Radhakrishnan et al (2021) | EA | PM_2.5_ | HR=0.75 (0.69–0.82) for Windsor region | μg/m³ | NR | 3.03% | HR=0.75 (0.69–0.82) | No |
|  |  |  | HR=0.71 (0.63–0.81) for London–Middlesex region |  |  |  | HR=0.71 (0.63–0.81) |  |
| Faraji et al (2024) | EA | PM_2.5_ | OR=0.99 (0.96–1.02) | μg/m³ | NR | NA | OR=0.99 (0.96–1.02) | No |
|  | CA | PM_2.5_ | OR=1.00 (0.98–1.03) |  |  |  | OR=1.00 (0.98–1.03) |  |
| Zanobetti et al (2024) | EA | PM_2.5_ | OR=1.18 (0.97–1.46) for first year of life | μg/m³ | 3.4 μg/m³ | NA | OR=1.63 (0.89–2.97) | Yes |
|  |  |  | OR=1.25 (1.01–1.54) for mean of year 1–2 * |  |  |  | OR=1.93 (1.04–3.58) |  |
|  |  |  | OR=1.30 (1.03–1.65) for mean of year 1–3 * |  |  |  | OR=2.16 (1.08–4.33) |  |
| Qiu et al (2024) | CA | PM_2.5_ | OR=1.252 (1.049–1.495) | μg/m³ | NR | NA | OR=1.252 (1.049–1.495) | No |
| Wang et al (2025) | CA | PM_2.5_ | HR=1.19 (1.10–1.28) | μg/m³ | 6.17 μg/m³ | 13.90% | HR=1.19 (1.10–1.28) | No |
| Wang et al (1999) | CA | PM_10_ | OR=1.00 (0.96–1.05) | μg/m³ | NR | NA | OR=1.00 (0.96–1.05) | No |
| Kuo et al (2002) | CA | PM_10_ | OR=0.947 (0.640–1.401) for ≧65.9μg/m^3^ | μg/m³ | NR | NA | OR=0.947 (0.640–1.401) | No |
| Ho et al (2007) | CA | PM_10_ | OR=0.993 (0.990–0.997) for female * | μg/m³ | NR | NA | OR=0.993 (0.990–0.997) | No |
| Sahsuvaroglu et al (2009) | EA | PM_10_ | OR=1.044 (0.891–1.225) for girls without hayfever | μg/m³ | 1μg/m³ | NA | OR=1.54 (0.31–7.56) | Yes |
| Anderson et al (2010) | EA | PM_10_ | OR=0.94 (0.87–1.01) | μg/m³ | 10 µg/m³ | NA | OR=0.94 (0.87–1.01) | Yes |
| Gruzieva et al (2013) | CA | PM_10_ | OR=1.96 (1.08–3.53) for road traffic during the first year of life * | μg/m³ | 10 µg/m³ | NA | OR=1.96 (1.08–3.53) | Yes |
|  |  |  | OR=1.02 (0.68–1.54) for road traffic since the previous follow–up |  |  |  | OR=1.02 (0.68–1.54) |  |
| Mölter et al (2014) | CA | PM_10_ | OR=0.87 (0.55–1.38) | μg/m³ | 1μg/m³ | NA | OR=0.25 (0.00–24.7) | Yes |
| Gehring et al (2015) | EA | PM_10_ | OR=1.08 (0.77–1.51) for road traffic at the birth address | μg/m³ | 10 µg/m³ | NA | OR=1.08 (0.77–1.51) | Yes |
|  |  |  | OR=0.91 (0.75–1.11) for road traffic at the current address |  |  |  | OR=0.91 (0.75–1.11) |  |
|  | CA | PM_10_ | OR=1.10 (0.74–1.63) for road traffic at the birth address |  |  |  | OR=1.10 (0.74–1.63) |  |
|  |  |  | OR=1.03 (0.80–1.34) for road traffic at the current address |  |  |  | OR=1.03 (0.80–1.34) |  |
| Rosa et al (2016) | EA | PM_10_ | OR=1.12 (1.00–1.21) * | μg/m³ | 38 μg/m³ | NA | OR=1.03 (1.00–1.06) | Yes |
| He et al (2019) | EA | PM_10_ | OR=0.95 (0.89–1.02) for in 0–2 year | μg/m³ | NR | NA | OR=0.95 (0.89–1.02) | No |
|  |  |  | OR=0.95 (0.88–1.02) for in 3–8year |  |  |  | OR=0.95 (0.88–1.02) |  |
| Liu et al (2020) | EA | PM_10_ | OR=1.60 (1.38–1.86) * | μg/m³ | 17.09μg/m³ | NA | OR=1.32 (1.21–1.44) | Yes |
|  | CA | PM_10_ | OR=1.65 (1.34–2.03) * |  |  |  | OR=1.34 (1.19–1.51) |  |
| Kuiper et al (2021) | CA | PM_10_ | OR=1.90 (1.06–3.41) * | μg/m³ | 10 µg/m³ | NA | OR=1.90 (1.06–3.41) | Yes |
| Faraji et al (2024) | EA | PM_10_ | OR=0.99 (0.98–1.00) | μg/m³ | NR | NA | OR=0.99 (0.98–1.00) | No |
|  | CA | PM_10_ | OR=0.99 (0.98–1.00) |  |  |  | OR=0.99 (0.98–1.00) |  |
| Wang et al (1999) | CA | CO | OR=1.15 (1.10–1.20) * | ppm | ≧0.80ppm | NA | OR=1.15 (1.10–1.20) | Yes |
| Lin et al (2001) | CA | CO | OR=1.10 (1.03–1.16) for 0.75–0.99 ppm * | ppm | 0.75–0.99 | NA | OR=1.10 (1.03–1.16) | Yes |
|  |  |  | OR=1.30 (1.18–1.42) for ≥ 1.0 ppm * |  | ≥ 1.0 ppm |  | OR=1.30 (1.18–1.42) |  |
| Delfino et al (2003) | CA | CO | OR=1.22 (0.43–3.43) for 1–hour max | ppb | 7.23ppb | NA | OR=1.22 (0.43–3.43) | Yes |
|  |  |  | OR=0.96 (0.27–3.38) for 8–hour max |  | 4.85ppb |  | OR=0.96 (0.27–3.38) |  |
| Ho et al (2007) | CA | CO | OR=1.984 (1.536–2.561) for female * | ppm | NR | NA | OR=1.984 (1.536–2.561) | Yes |
|  |  |  | OR=1.780 (1.377–2.302) for male * |  |  |  | OR=1.780 (1.377–2.302) |  |
| Faraji et al (2024) | EA | CO | OR=1.09 (0.54–2.27) | ppm | 1.63 ppm | NA | OR=1.09 (0.54–2.27) | Yes |
|  | CA | CO | OR=1.02 (0.59–1.75) |  |  | NA | OR=1.02 (0.59–1.75) |  |
| Dockery et al (1989) | EA | NO_2_ | OR=0.60 (0.30–0.90) | ppb | 16.1ppb | NA | OR=0.84 (0.70–1.01) | Yes |
| Wang et al (1999) | CA | NO_2_ | OR=1.08 (1.04–1.13) * | ppm | NR | NA | OR=1.08 (1.04–1.13) | No |
| Shima et al (2000) | CA | NO_2_ | OR=2.10 (1.10–4.75) * | ppb | 10ppb | NA | OR=1.48 (1.01–2.19) | Yes |
| Kuo et al (2002) | CA | NO_2_ | OR=1.692 (1.155–2.480) for ≧ 0.023 ppm * | ppm | NR | NA | OR=1.692 (1.155–2.480) | No |
| Delfino et al (2003) | CA | NO_2_ | OR=8.13 (1.52–43.4) for 1–hour max * | ppb | 7.14ppb | NA | OR=4.76 (1.37–16.55) | Yes |
|  |  |  | OR=7.14 (1.66–30.7) for 8–hour max * |  | 5.99ppb |  | OR=5.72 (1.57–20.86) |  |
| Mi et al (2006) | CA | NO_2_ | OR=1.23 (0.87–1.73) for asthma attacks | μg/m³ | 10 µg/m³ | NA | OR=1.23 (0.87–1.73) | Yes |
|  |  |  | OR=1.44 (1.06–1.95) for asthma attacks and CA medication * |  |  |  | OR=1.44 (1.06–1.95) |  |
| Solé et al (2007) | CA | NO_2_ | OR=1.87 (1.47–2.38) for 81.0 µg/m^3^ * | μg/m³ | NR | NA | OR=1.87 (1.47–2.38) | No |
|  |  |  | OR=0.93 (0.70–1.23) for 39.0 µg/m^3^ |  |  |  | OR=0.93 (0.70–1.23) |  |
|  |  |  | OR=0.97 (0.74–1.28) for 33.0 µg/m^3^ |  |  |  | OR=0.97 (0.74–1.28) |  |
|  |  |  | OR=1.57 (1.22–2.02) for 34.5 µg/m^3^ * |  |  |  | OR=1.57 (1.22–2.02) |  |
| Zhao et al (2008) | CA | NO_2_ | OR=0.66 (0.37–1.10) | μg/m³ | 10 µg/m³ | NA | OR=0.66 (0.37–1.10) | Yes |
| Sahsuvaroglu et al (2009) | EA | NO_2_ | OR=1.271 (0.992–1.627) for girls without hayfever * | ppb | 1ppb | NA | OR=3.58 (0.96–13.31) | Yes |
| Jerrett et al (2011) | EA | NO_2_ | HR=1.29 (1.11–1.49) for in fall winter * | ppb | 6.2 ppb | 1.42% | OR=1.27 (1.12–1.44) | Yes |
|  |  |  | HR=1.27 (1.03–1.57) for in summer * |  |  |  | OR=1.25 (1.05–1.50) |  |
|  |  |  | HR=1.29 (1.07–1.56) for in annual * |  |  |  | OR=1.27 (1.08–1.49) |  |
| Fuertes et al (2013) | CA | NO_2_ | OR=0.89 (0.73–1.08) | μg/m³ | 6.1μg/m³ | NA | OR=0.83 (0.60–1.14) | Yes |
| Mölter et al (2014) | CA | NO_2_ | OR=1.05 (0.87–1.25) | μg/m³ | 1μg/m³ | NA | OR=1.63 (0.27–9.97) | Yes |
| Gehring et al (2015) | EA | NO_2_ | OR=1.13 (1.02–1.25) for road traffic at the birth address * | μg/m³ | 10 µg/m³ | NA | OR=1.13 (1.02–1.25) | Yes |
|  |  |  | OR=1.03 (0.88–1.19) for road traffic at the current address |  |  |  | OR=1.03 (0.88–1.19) |  |
|  | CA | NO_2_ | OR=1.06 (0.88–1.26) for road traffic at the birth address |  |  |  | OR=1.06 (0.88–1.26) |  |
|  |  |  | OR=1.04 (0.93–1.16) for road traffic at the current address |  |  |  | OR=1.04 (0.93–1.16) |  |
| Yang et al (2016) | CA | NO_2_ | OR=1.08 (0.97–1.21) | μg/m³ | 8.4µg/m³ | NA | OR=1.10 (0.96–1.25) | Yes |
| Greenberg et al (2016) | EA | NO_2_ | OR=1.301 (1.187–1.426) for 14.1–27.2 μg/m^3^ | μg/m³ | NR | NA | OR=1.301 (1.187–1.426) | No |
|  |  |  | OR=1.391 (1.264–1.531) for 27.2–43.2μg/m^3^ |  |  |  | OR=1.391 (1.264–1.531) |  |
| Greenberg et al (2017) | EA | NO_2_ | OR=1.01 (1.01–1.01) for arithmetic mean of average concentration | μg/m³ | NR | NA | OR=1.01 (1.01–1.01) | No |
| He et al (2019) | EA | NO_2_ | OR=1.03 (0.96–1.09) for in 0–2 year | μg/m³ | NR | NA | OR=1.03 (0.96–1.09) | No |
|  |  |  | OR=1.01 (0.94–1.08) for in 3–8year |  |  |  | OR=1.01 (0.94–1.08) |  |
| Liu et al (2020) | EA | NO_2_ | OR=1.58 (1.36–1.84) * | μg/m³ | 7.70μg/m³ | NA | OR=1.81 (1.49–2.20) | Yes |
|  | CA | NO_2_ | OR=1.64 (1.34–2.02) * |  |  |  | OR=1.90 (1.46–2.48) |  |
| To et al (2020) | EA | NO_2_ | OR=1.17 (1.05–1.31) for at birth exposure | μg/m³ | 27.03μg/m³ | NA | OR=1.06 (1.02–1.10) | Yes |
|  |  |  | OR=1.12 (1.00–1.26) for at 3-year exposure |  | 26.53μg/m³ |  | OR=1.04 (1.00–1.09) |  |
| Kuiper et al (2021) | CA | NO_2_ | OR=1.29 (1.02–1.63) * | μg/m³ | 10 µg/m³ | NA | OR=1.29 (1.02–1.63) | Yes |
| Radhakrishnan et al (2021) | EA | NO_2_ | HR=0.70 (0.62–0.81) for Windsor region | ppb | NR | 3.03% | HR=0.70 (0.62–0.81) | No |
|  |  |  | HR=0.65 (0.60–0.69) for London–Middlesex region |  |  |  | HR=0.65 (0.60–0.69) |  |
| Faraji et al (2024) | EA | NO_2_ | OR=0.99 (0.97–1.01) | ppb | NR | NA | OR=0.99 (0.97–1.01) | No |
|  | CA | NO_2_ | OR=0.99 (0.98–1.01) |  |  |  | OR=0.99 (0.98–1.01) |  |
| Zanobetti et al (2024) | EA | NO_2_ | OR=1.23 (1.02–1.47) for first year of life * | μg/m³ | 6.1μg/m³ | NA | OR=1.40 (1.04–1.89) | Yes |
|  |  |  | OR=1.30 (1.08–1.57) for mean of year 1–2 * |  |  |  | OR=1.54 (1.13–2.09) |  |
|  |  |  | OR=1.31 (1.07–1.60) for mean of year 1–3 * |  |  |  | OR=1.56 (1.12–2.17) |  |
| Wang et al (2025) | CA | NO_2_ | HR=1.19 (1.05–1.34) | ppb | 15.37 ppb | 15.50% | HR=1.19 (1.05–1.34) | No |
| Dockery et al (1989) | EA | O_3_ | OR=1.90 (1.00–3.40) | ppb | 19.8 ppb | NA | OR=1.18 (1.01–1.38) | Yes |
| Wang et al (1999) | CA | O_3_ | OR=1.11 (1.07–1.15) * | ppm | NR | NA | OR=1.11 (1.07–1.15) | No |
| Kuo et al (2002) | CA | O_3_ | OR=0.750 (0.318–1.769) for ≧ 23 ppb | ppb | NR | NA | OR=0.750 (0.318–1.769) | No |
| Delfino et al (2003) | CA | O_3_ | OR=0.60 (0.09–3.87) for 1–hour max | ppb | 25.5 ppb | NA | OR=0.90 (0.62–1.31) | Yes |
|  |  |  | OR=0.50 (0.08–3.23) for 8–hour max |  | 16.8 ppb |  | OR=0.81 (0.46–1.42) |  |
| Mi et al (2006) | CA | O_3_ | OR=0.37 (0.12–1.21) for asthma attacks | μg/m³ | 10 µg/m³ | NA | OR=0.37 (0.12–1.21) | Yes |
|  |  | O_3_ | OR=0.54 (0.21–1.41) for asthma attacks and CA medication |  |  |  | OR=0.54 (0.21–1.41) |  |
| Ho et al (2007) | CA | O_3_ | OR=1.015 (1.001–1.029) for male * | ppb | NR | NA | OR=1.015 (1.001–1.029) | No |
| Zhao et al (2008) | CA | O_3_ | OR=0.50 (0.11–2.28) | μg/m³ | 10 µg/m³ | NA | OR=0.50 (0.11–2.28) | Yes |
| Sahsuvaroglu et al (2009) | EA | O_3_ | OR=0.998 (0.691–1.440) for girls without hayfever | ppb | 1ppb | NA | OR=0.99 (0.15–6.43) | Yes |
| Fuertes et al (2013) | CA | O_3_ | OR=1.20 (0.98–1.48) | μg/m³ | 5.2 µg/m³ | NA | OR=1.42 (0.96–2.11) | Yes |
| To et al (2020) | EA | O_3_ | OR=1.22 (1.04–1.43) for at birth exposure | ppb | 43.26ppb | NA | OR=1.02 (1.00–1.04) | Yes |
|  |  |  | OR=1.13 (0.97–1.31) for at 3-year exposure |  | 43.37 ppb |  | OR=1.01 (1.00–1.03) |  |
| Kuiper et al (2021) | CA | O_3_ | OR=2.00 (1.11–3.58) * | μg/m³ | 10 µg/m³ | NA | OR=2.00 (1.11–3.58) | Yes |
| Radhakrishnan et al (2021) | EA | O_3_ | HR=0.72 (0.67–0.78) for Windsor region | ppb | NR | 3.03%, | HR=0.72 (0.67–0.78) | No |
|  |  |  | HR=0.65 (0.60–0.69) for London–Middlesex region |  |  |  | HR=0.65 (0.60–0.69) |  |
| Faraji et al (2024) | EA | O_3_ | OR=0.86 (0.74–1.00) * | ppb | NR | NA | OR=0.86 (0.74–1.00) | No |
|  | CA | O_3_ | OR=0.79 (0.70–0.89) * |  |  |  | OR=0.79 (0.70–0.89) |  |
| Wang et al (2025) | CA | O_3_ | HR=1.11 (1.01–1.22) | ppb | 6.87 ppb | 15.50% | HR=1.11 (1.01–1.22) | No |
| Dockery et al (1989) | EA | SO_2_ | OR=0.60 (0.30–1.20) | ppb | 24.3ppb | NA | OR=0.92 (0.83–1.03) | Yes |
| Wang et al (1999) | CA | SO_2_ | OR=0.98 (0.95–1.02) | ppm | NR | NA | OR=0.98 (0.95–1.02) | No |
| Kuo et al (2002) | CA | SO_2_ | OR=1.198 (0.674–2.127) for ≧ 0.005 ppm | ppm | NR | NA | OR=1.198 (0.674–2.127) | No |
| Delfino et al (2003) | CA | SO_2_ | OR=2.36 (1.16–4.81) for 1–hour max * | ppb | 7.33ppb | NA | OR=1.56 (1.08–2.27) | Yes |
|  |  |  | OR=1.91 (1.06–3.43) for 8–hour max * |  | 4.97ppb |  | OR=1.64 (1.05–2.58) |  |
| Solé et al (2007) | CA | SO_2_ | OR=2.01 (1.56–2.60) for 7.0 µg/m^3^ * | μg/m³ | NR | NA | OR=2.01 (1.56–2.60) | No |
|  |  |  | OR=1.04 (0.78–1.40) for 16.0 µg/m^3^ |  |  | NA | OR=1.04 (0.78–1.40) |  |
|  |  |  | OR=1.08 (0.81–1.42) for 40.5 µg/m^3^ |  |  | NA | OR=1.08 (0.81–1.42) |  |
| Zhao et al (2008) | CA | SO_2_ | OR=0.97 (0.70–1.35) | μg/m³ | 100μg/m³ | NA | OR=1.00 (0.96–1.03) | Yes |
| Sahsuvaroglu et al (2009) | EA | SO_2_ | OR=1.246 (0.802–1.934) for girls without hayfever | ppb | 1ppb | NA | OR=2.32 (0.43–12.43) | Yes |
| Chiang et al (2016) | EA | SO_2_ | HR=1.29 (0.91–1.83) for high SO_2_ exposure in 1999–2010 | ppb | NR | NA | HR=1.29 (0.91–1.83) | No |
| Greenberg et al (2016) | EA | SO_2_ | OR=1.070 (1.016–1.126) for 6.7–13.3 μg/m^3^ | μg/m³ | NR | NA | OR=1.070 (1.016–1.126) | No |
|  |  |  | OR=1.369 (1.266–1.481) for 13.3–592.7μg/m^3^ |  |  |  | OR=1.369 (1.266–1.481) |  |
| Greenberg et al (2017) | EA | SO_2_ | OR=1.00 (1.00–1.00) for arithmetic mean of SO_2_ | mg/m³ | NR | NA | OR=1.00 (1.00–1.00) | No |
| He et al (2019) | EA | SO_2_ | OR=0.96 (0.89–1.03) for in 0–2 year | μg/m³ | NR | NA | OR=0.96 (0.89–1.03) | No |
|  |  |  | OR=0.98 (0.91–1.05) for in 3–8year |  |  |  | OR=0.98 (0.91–1.05) |  |
| Radhakrishnan et al (2021) | EA | SO_2_ | HR=0.80 (0.68–0.93) for Windsor region | ppb | NR | 3.03%, | HR=0.80 (0.68–0.93) | No |
|  |  |  | HR=0.81 (0.60–1.09) for London–Middlesex region |  |  |  | HR=0.81 (0.60–1.09) |  |
| Faraji et al (2024) | EA | SO_2_ | OR=0.92 (0.81–1.05) | ppb | NR | NA | OR=0.92 (0.81–1.05) | No |
|  | CA | SO_2_ | OR=0.96 (0.87–1.07) |  |  |  | OR=0.96 (0.87–1.07) |  |
| Duhme et al (1998) | CA | TRAP | POR=1.06 (0.83–1.36) for seldom ^a^ (WQ: Münster) | NA | NA | 13.10% | POR=1.06 (0.83–1.36) | No |
|  |  |  | POR=1.10 (0.90–1.35) for seldom ^a^ (VQ: Münster) |  |  | 19.90% | POR=1.10 (0.90–1.35) |  |
|  |  |  | POR=1.05 (0.63–1.73) for seldom ^a^ (WQ: Greifswald) |  |  | 12.80% | POR=1.05 (0.63–1.73) |  |
|  |  |  | POR=1.27 (0.82–1.97) for seldom ^a^ (VQ: Greifswald) |  |  | 20.50% | POR=1.27 (0.82–1.97) |  |
|  |  |  | POR=1.68 (1.28–2.21) for frequent or constant ^a^ (WQ: Münster) * |  |  | 13.10% | POR=1.68 (1.28–2.21) |  |
|  |  |  | POR=1.60 (1.26–2.02) for frequent or constant ^a^ (VQ: Münster) * |  |  | 19.90% | POR=1.60 (1.26–2.02) |  |
|  |  |  | POR=1.46 (0.88–2.41) for frequent or constant ^a^ (WQ: Greifswald) |  |  | 12.80% | POR=1.46 (0.88–2.41) |  |
|  |  |  | POR=1.72 (1.11–2.67) for frequent or constant ^a^ (VQ: Greifswald) * |  |  | 20.50% | POR=1.72 (1.11–2.67) |  |
| Del–Rio–Navarro et al (2006) | CA | TRAP | OR=1.206 (1.066–1.363) for boy * | NA | NA | NA | OR=1.206 (1.066–1.363) | Yes |
|  |  |  | OR=1.136 (1.008–1.280) for girls * |  |  |  | OR=1.136 (1.008–1.280) |  |
| Brunekreef et al (2009) | EA | TRAP | OR=1.18 (1.08–1.28) for high frequently ^a^ * | NA | NA | NA | OR=1.18 (1.08–1.28) | Yes |
|  |  |  | OR=1.08 (1.00–1.17) for medium frequently ^a^ * |  |  |  | OR=1.08 (1.00–1.17) |  |
|  |  |  | OR=1.01 (0.94–1.09) for low frequently ^a^ |  |  |  | OR=1.01 (0.94–1.09) |  |
|  | CA | TRAP | OR=1.53 (1.36–1.72) for high frequently ^a^ * |  |  |  | OR=1.53 (1.36–1.72) |  |
|  |  |  | OR=1.26 (1.13–1.41) for medium frequently ^a^ * |  |  |  | OR=1.26 (1.13–1.41) |  |
|  |  |  | OR=1.07 (0.97–1.18) for low frequently ^a^ |  |  |  | OR=1.07 (0.97–1.18) |  |
| Musharrafieh et al (2009) | CA | TRAP | OR=0.90 (0.70–1.10) for continuously during daytime ^a^ | NA | NA | NA | OR=0.90 (0.70–1.10) | Yes |
|  |  |  | OR=1.30 (1.00–1.60) for all day long ^a^ |  |  |  | OR=1.30 (1.00–1.60) |  |
| Kasznia–Kocot et al (2010) | EA | TRAP | OR=1.93 (1.09–3.41) * | NA | NA | NA | OR=1.93 (1.09–3.41) | Yes |
| Cibella et al (2011) | CA | TRAP | OR=1.84 (1.14–2.95) for frequent or constant ^a^ * | NA | NA | NA | OR=1.84 (1.14–2.95) | Yes |
| Gonzalez–barcala et al (2013) | EA | TRAP | OR=1.09 (0.82–1.44) for boys seldom ^a^ | NA | NA | NA | OR=1.09 (0.82–1.44) | Yes |
|  |  |  | OR=1.13 (0.83–1.52) for boys frequent ^a^ |  |  |  | OR=1.13 (0.83–1.52) |  |
|  |  |  | OR=1.01 (0.64–1.60) for boys constant ^a^ |  |  |  | OR=1.01 (0.64–1.60) |  |
|  |  |  | OR=1.30 (0.91–1.87) for girls seldom ^a^ |  |  |  | OR=1.30 (0.91–1.87) |  |
|  |  |  | OR=1.09 (0.75–1.59) for girls frequent ^a^ |  |  |  | OR=1.09 (0.75–1.59) |  |
|  |  |  | OR=1.18 (0.71–1.97) for girls constant ^a^ |  |  |  | OR=1.18 (0.71–1.97) |  |
|  | CA | TRAP | OR=0.89 (0.64–1.25) for boys seldom ^a^ |  |  |  | OR=0.89 (0.64–1.25) |  |
|  |  |  | OR=1.01 (0.71–1.45) for boys frequent ^a^ |  |  |  | OR=1.01 (0.71–1.45) |  |
|  |  |  | OR=0.98 (0.57–1.69) for boys constant ^a^ |  |  |  | OR=0.98 (0.57–1.69) |  |
|  |  |  | OR=1.02 (0.70–1.48) for girl seldom ^a^ |  |  |  | OR=1.02 (0.70–1.48) |  |
|  |  |  | OR=1.12 (0.76–1.65) for girls frequent ^a^ |  |  |  | OR=1.12 (0.76–1.65) |  |
|  |  |  | OR=1.17 (0.69–1.99) for girls constant ^a^ |  |  |  | OR=1.17 (0.69–1.99) |  |
| Oluwole et al (2013) | EA | TRAP | OR=1.01 (0.91–1.68) for seldom ^a^ | NA | NA | NA | OR=1.01 (0.91–1.68) | Yes |
|  |  |  | OR=1.77 (1.04–3.01) for frequent ^a^ |  |  |  | OR=1.77 (1.04–3.01) |  |
|  |  |  | OR=0.94 (0.54–1.63) for almost ^a^ |  |  |  | OR=0.94 (0.54–1.63) |  |
| Gomes de Luna Mde et al (2015) | CA | TRAP | OR=1.41 (0.79–2.52) | NA | NA | NA | OR=1.41 (0.79–2.52) | Yes |
| Hedman et al (2015) | EA | TRAP | HR=1.10 (0.81–1.50) for living within 200 m from a heavily traffic road or much used bus stop | NA | NA | 7.20% | OR=1.21 (0.81–1.50) | Yes |
|  | CA | TRAP | HR=1.07 (0.75–1.53) for living within 200 m from a heavily traffic road or much used bus stop |  |  | 6.30% | OR=1.08 (0.75–1.53) |  |
| Chiang et al (2016) | EA | TRAP | HR=1.23 (0.87–1.73) for in 1999–2010 | NA | NA | 20.20% |  | No |
| Bowatte et al (2016) | CA | TRAP | OR=1.02 (0.86–1.22) for cumulative lengths of major roads in 150 m buffer of residence during 1 yr of life in 12-year child | NA | NA | NA | OR=1.02 (0.86–1.22) | Yes |
|  |  |  | OR=1.02 (0.86–1.21) for cumulative lengths of major roads in 150 m buffer of residence during 1 yr of life in 18-year child |  |  |  | OR=1.02 (0.86–1.21) |  |
|  |  |  | OR=0.78 (0.36–1.70) for living ≤150 m from a freeway or highway during 1 yr of life in 12-year child |  |  |  | OR=0.78 (0.36–1.70) |  |
|  |  |  | OR=1.24 (0.65–2.36) for living ≤150 m from a freeway or highway during 1 yr of life in 18-year child |  |  |  | OR=1.24 (0.65–2.36) |  |
| Arrais et al (2017) | CA | TRAP | OR=1.236 (0.85–1.79) for seldom ^a^ | NA | NA | NA | OR=1.236 (0.85–1.79) | Yes |
|  |  |  | OR=1.36 (0.94–1.97) for frequently ^a^ |  |  |  | OR=1.36 (0.94–1.97) |  |
|  |  |  | OR=1.56 (1.05–2.34) for almost ^a^ * |  |  |  | OR=1.56 (1.05–2.34) |  |
| Skrzypek et al (2019) | EA | TRAP | OR=2.16 (1.12–4.15) for living in the vicinity of a main road * | NA | NA | NA | OR=2.16 (1.12–4.15) | Yes |
|  |  |  | OR=2.31 (1.22–4.39) for near the place of residence * |  |  |  | OR=2.31 (1.22–4.39) |  |
| Rutter et al (2020) | CA | TRAP | OR=1.14 (1.09–1.18) for heavy truck traffic * | NA | NA | NA | OR=1.14 (1.09–1.18) | Yes |
| Ahmetaj et al (2023) | CA | TRAP | OR=0.84 (0.16–4.51) for seldom in Ferizaj ^a^ | NA | NA | NA | OR=0.84 (0.16–4.51) | Yes |
|  |  |  | OR=1.64 (0.30–8.90) for frequently in Ferizaj ^a^ |  |  |  | OR=1.64 (0.30–8.90) |  |
|  |  |  | OR=1.35 (0.23–7.92) for almost in Ferizaj ^a^ |  |  |  | OR=1.35 (0.23–7.92) |  |
|  |  |  | OR=2.41 (0.52–11.2) for seldom in Gjakova ^a^ |  |  |  | OR=2.41 (0.52–11.2) |  |
|  |  |  | OR=3.27 (0.68–15.7) for frequently in Gjakova ^a^ |  |  |  | OR=3.27 (0.68–15.7) |  |
|  |  |  | OR=1.82 (0.30–11.1) for almost the in Gjakova ^a^ |  |  |  | OR=1.82 (0.30–11.1) |  |
|  |  |  | OR=0.68 (0.28–1.63) for seldom in Gjilan ^a^ |  |  |  | OR=0.68 (0.28–1.63) |  |
|  |  |  | OR=1.41 (0.57–3.52) for frequently in Gjilan ^a^ |  |  |  | OR=1.41 (0.57–3.52) |  |
|  |  |  | OR=1.02 (0.34–3.02) for almost in Gjilan ^a^ |  |  |  | OR=1.02 (0.34–3.02) |  |
|  |  |  | OR=1.33 (0.66–2.71) for seldom in Peja ^a^ |  |  |  | OR=1.33 (0.66–2.71) |  |
|  |  |  | OR=1.21 (0.56–2.60) for frequently in Peja ^a^ |  |  |  | OR=1.21 (0.56–2.60) |  |
|  |  |  | OR=1.29 (0.54–3.10) for almost the in Peja ^a^ |  |  |  | OR=1.29 (0.54–3.10) |  |
|  |  |  | OR=0.81 (0.29–2.29) for seldom in Prishtina ^a^ |  |  |  | OR=0.81 (0.29–2.29) |  |
|  |  |  | OR=1.57 (0.55–4.50) for frequently in Prishtina ^a^ |  |  |  | OR=1.57 (0.55–4.50) |  |
|  |  |  | OR=0.73 (0.20–2.59) for almost in Prishtina ^a^ |  |  |  | OR=0.73 (0.20–2.59) |  |
|  |  |  | OR=0.92 (0.50–1.69) for seldom in Prizren ^a^ |  |  |  | OR=0.92 (0.50–1.69) |  |
|  |  |  | OR=0.88 (0.45–1.69) for frequently in Prizren ^a^ |  |  |  | OR=0.88 (0.45–1.69) |  |
|  |  |  | OR=0.80 (0.33–1.96) for almost in Prizren ^a^ |  |  |  | OR=0.80 (0.33–1.96) |  |
| Mphahlele et al (2023) | CA | TRAP | OR=1.423 (1.111–1.822) for truck frequency outside the home * | NA | NA | NA | OR=1.423 (1.111–1.822) | Yes |
| Rathogwa–Takalani et al (2024) | EA | TRAP | OR=1.13 (0.74–1.73) for seldom ^a^ | NA | NA | NA | OR=1.13 (0.74–1.73) | Yes |
|  |  |  | OR=1.66 (0.99–2.00) for frequently ^a^ |  |  |  | OR=1.66 (0.99–2.00) |  |
|  |  |  | OR=1.64 (0.99–1.94) for almost ^a^ |  |  |  | OR=1.64 (0.99–1.94) |  |
| Jafarinodoshan et al (2024) | EA | TRAP | OR=1.25 (0.98–1.59) for seldom ^a^ | NA | NA | NA | OR=1.25 (0.98–1.59) | Yes |
|  |  |  | OR=1.89 (1.39–2.57) for frequently ^a^ |  |  |  | OR=1.89 (1.39–2.57) |  |
|  |  |  | OR=2.19 (1.33–3.62) for almost ^a^ |  |  |  | OR=2.19 (1.33–3.62) |  |
| Malamardi et al (2024) | EA | TRAP | OR=1.638 (0.952–2.817) for passing of trucks near residence | NA | NA | NA | OR=1.638 (0.952–2.817) | Yes |

PR: percentile range; HO: health outcome; EA: ever asthma; CA: current asthma; NA: not applicable; NR: not reported; WQ: written questionnaire; VQ: video questionnaire; ^a^ The frequency of buses/trucks pass by house, heavy truck traffic, density of road traffic, and residential proximity to major roadways (seldom, frequent, or constant); OR: odds ratio; HR: hazard ratio; POR: prevalence odds ratio; PM_2.5_: particulate matter with diameter ≤ 2.5 micrometers ; PM_10_: particulate matter with diameter ≤ 10 micrometers; CO: carbon monoxide; NO_2_: nitrogen dioxide; O_3_: ozone; SO_2_: sulfur dioxide; TRAP: traffic-related air pollution; μg/m³: microgram per cubic meter; ppm: parts per million; ppb: parts per billion; * p value <0.05.
